# Investigation of the BERT model on nucleotide sequences with non-standard pre-training and evaluation of different k-mer embeddings

**DOI:** 10.1093/bioinformatics/btad617

**Published:** 2023-10-10

**Authors:** Yao-zhong Zhang, Zeheng Bai, Seiya Imoto

**Affiliations:** Division of Health Medical Intelligence, Human Genome Center, The Institute of Medical Science, The University of Tokyo, Minato-ku, Tokyo 108-8639, Japan; Division of Health Medical Intelligence, Human Genome Center, The Institute of Medical Science, The University of Tokyo, Minato-ku, Tokyo 108-8639, Japan; Division of Health Medical Intelligence, Human Genome Center, The Institute of Medical Science, The University of Tokyo, Minato-ku, Tokyo 108-8639, Japan

## Abstract

**Motivation:**

In recent years, pre-training with the transformer architecture has gained significant attention. While this approach has led to notable performance improvements across a variety of downstream tasks, the underlying mechanisms by which pre-training models influence these tasks, particularly in the context of biological data, are not yet fully elucidated.

**Results:**

In this study, focusing on the pre-training on nucleotide sequences, we decompose a pre-training model of Bidirectional Encoder Representations from Transformers (BERT) into its embedding and encoding modules to analyze what a pre-trained model learns from nucleotide sequences. Through a comparative study of non-standard pre-training at both the data and model levels, we find that a typical BERT model learns to capture overlapping-consistent k-mer embeddings for its token representation within its embedding module. Interestingly, using the k-mer embeddings pre-trained on random data can yield similar performance in downstream tasks, when compared with those using the k-mer embeddings pre-trained on real biological sequences. We further compare the learned k-mer embeddings with other established k-mer representations in downstream tasks of sequence-based functional prediction. Our experimental results demonstrate that the dense representation of k-mers learned from pre-training can be used as a viable alternative to one-hot encoding for representing nucleotide sequences. Furthermore, integrating the pre-trained k-mer embeddings with simpler models can achieve competitive performance in two typical downstream tasks.

**Availability and implementation:**

The source code and associated data can be accessed at https://github.com/yaozhong/bert_investigation.

## 1 Introduction

Pre-training with the transformer architecture (Vaswani *et al.* 2017) has achieved remarkable results across a variety of research fields, including natural language processing (Devlin *et al.* 2018) and computer vision (Dosovitskiy *et al.* 2020). In the realm of computational biology, this methodology has been used to learn representations for amino acid sequences (Jumper *et al.* 2021, Rao *et al.* 2021,  Elnaggar et al., 2022) and nucleotide sequences (Ji *et al.* 2021). Given the scarcity of labeled data, the pre-training and fine-tuning paradigm leverages abundant unlabeled data to learn general representations. These representations are subsequently refined via fine-tuning, utilizing a limited dataset of labeled data specific to the target downstream task. While this training paradigm has resulted in significant performance improvements in a range of downstream applications, the underlying mechanisms that contribute to the efficacy of pre-trained models remain incompletely understood. Furthermore, current research tends to focus on model architectures that are reminiscent of those used in natural language modeling tasks. The unique characteristics inherent to biological sequences have been less extensively explored.

In this study, we focus on nucleotide sequences and aim to understand what a pre-trained Bidirectional Encoder Representations from Transformers (BERT) model (Devlin *et al.* 2018, Ji *et al.* 2021) learns during pre-training and how the acquired knowledge influences performance in downstream tasks. Previous work (Ji *et al.* 2021) used attention weights to interpret regions that contribute to the predictive outcomes for specific input sequences. However, non-sequence-specific token embeddings learned by the pre-trained model have not been extensively investigated. Here, we concentrate on the non-sequence-specific lower-level token embeddings to provide a global interpretation (Novakovsky *et al.* 2023) of the pre-trained model. We decompose the BERT model into its embedding and encoding modules. We employ a non-standard pre-training method by incorporating randomness at both the data and model levels for comparative analysis. In addition to evaluating pre-trained BERT models, we investigate the use of different k-mer embeddings in the sequence-based functional prediction tasks, including TATA promoter prediction and transcription factor binding site (TFBS) prediction. We demonstrate potential applications of a BERT model pre-trained on randomly generated sequences. These applications encompass its utility as a dense k-mer token representation and as a model weight initializer to accelerate the pre-training process.

## 2 Materials and methods

To understand what a BERT model captures during the pre-training phase, we decomposed a standard BERT model into embedding and encoding modules. The embedding module comprises embeddings that represent tokens, token types, and positions. The encoding module employs the encoder component of the transformer model (Vaswani *et al.* 2017), incorporating multi-head attention and feed-forward layers. To analyze the pre-training of the BERT model on nucleotide sequences, we utilized DNABERT (Ji *et al.* 2021). (In the subsequent sections, the terms “BERT” and “DNABERT” are used interchangeably, referring to the BERT model applied to nucleotide sequences.)

**Figure 1.**
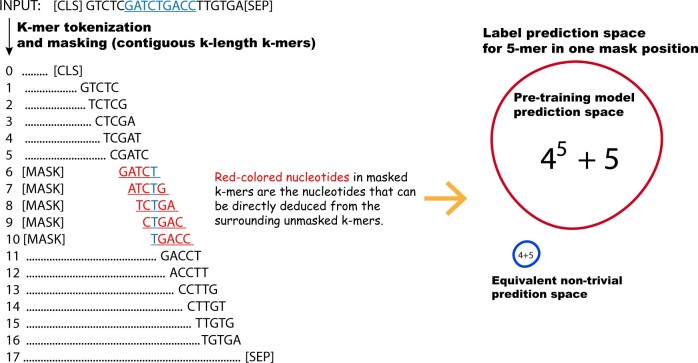
K-mer token masking strategy used in DNABERT. A nucleotide sequence is first converted into an overlapping k-mer sequence, with special tokens inserted at both ends of the sequence. For pre-training, partial tokens are masked to train the model for their prediction. To preclude the trivial inference of a masked token from its immediately adjacent k-mers, DNABERT masks k contiguous k-mers. Compared with the prediction space of the non-trivially inferable nucleotides (those that cannot be directly deduced from adjacent k-mers), the original prediction space is appreciably larger.

During pre-training, the BERT model is trained to predict masked tokens. In the context of nucleotide sequences, DNABERT uses a strategy of masking *k* contiguous k-mers, to preclude trivial inference of the masked tokens based on their adjacent k-mers. This masking approach is exemplified in a 5-mer illustration shown in Fig. 1, where five contiguous 5-mers are masked during the pre-training phase. However, upon decomposing a k-mer to the nucleotide level, it becomes apparent that specific nucleotides within the masked k-mers may still be deducible from adjacent k-mers. As a consequence, for *k* contiguous masked k-mers, only a single nucleotide cannot be deduced from neighboring k-mers. The DNABERT model has a label search space of $4^{k}+5$ (with the five additional labels corresponding to special tokens used in BERT, e.g. [CLS], [PAD]). However, the search space for non-trivially inferable labels is reduced to 4+5. Based on this observation, the pre-training task of DNABERT can be divided into two subtasks: (i) Overlapping-consistent k-mer prediction for the contiguous masked k-mers. For instance, for two consecutive masked tokens, G**ATCT** and **ATCT**G, predictions maintaining consistent overlap (e.g. G**ATCT** and **ATCT**G) are preferable to inconsistent ones (e.g. G**ATCT** and **GTTT**G). (ii) Prediction of the non-trivially inferable nucleotide within k-mers. For different pre-training datasets, such as nucleotide sequences originating from different species, the first subtask remains data-independent. In contrast, the second subtask is data-dependent. Previous work (Liang *et al.* 2022) on predicting nucleotides from their surrounding nucleotides has demonstrated variable performance across different regions of human chromosomes. Specifically, repeat regions exhibit higher accuracy, whereas coding regions show lower accuracy, yet the overall accuracy exceeds 50%. Note that the encoding module constitutes a significant portion of the model parameters. Taking the 5-mer DNABERT model for instance, the encoding module accounts for ∼98.6% of the total model parameters (amounting to 85 million). This substantial number of model parameters enables the model to make overlapping-consistent k-mer predictions across a vast label search space during pre-training on nucleotide sequences.

**Figure 2.**
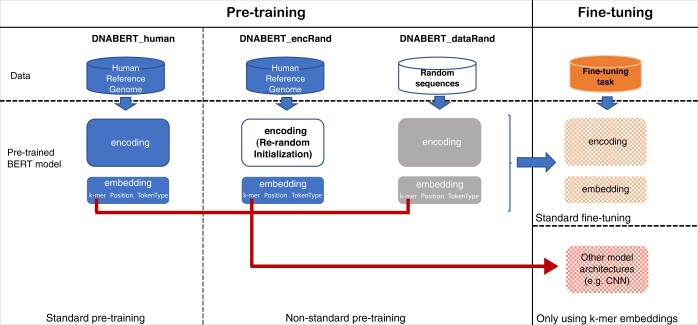
Methodology for decomposition and analysis of the DNABERT model. The model is decomposed into embedding and encoding modules. To investigate the DNABERT model, we conducted non-standard pre-training that incorporates randomness at both the data and model levels. At the data level, we generated nucleotide sequences randomly, comprising ∼3 billion bases, for comparison with the DNABERT pre-trained on the human reference genome. At the model level, we incorporated randomness into the encoding module through re-initializing the corresponding model weights. In the fine-tuning stage, in addition to the standard fine-tuning, we also assessed simpler neural network structures that solely utilize the learned k-mer embeddings.

To illustrate the learning outcomes of the BERT model applied to nucleotide sequences, we adopted a non-standard pre-training approach, introducing randomness at both the data and model levels for a comprehensive analysis of the DNABERT model. At the data level, we generated entirely random nucleotide sequences, comprising ∼3 billion bases, for comparative analysis with the DNABERT pre-trained on the human reference genome. With the exception of the pre-training data difference, we maintained all other pre-training settings consistent with DNABERT and trained a DNABERT model on the randomly generated nucleotide sequences. The contrastive analysis allows us to investigate the first subtask, given that the random sequences are devoid of biological information that can be used to predict context-dependent nucleotides. At the model level, we introduced randomness into the encoding module of DNABERT, as illustrated in Fig. 2. We re-initialized the learned model weights in the encoding module while retaining the embeddings from the pre-trained model. We further examined the “ablated” pre-trained models fine-tuned for downstream tasks, comparing them with other pre-trained models using the human reference genome and random sequences. These comparative assessments assist to analyze the learning outcomes of the pre-trained DNABERT model on nucleotide sequences. Furthermore, we extended our evaluation to include simpler model structures previously recognized as state-of-the-art. Specifically, we assessed DeePromoter (Oubounyt *et al.* 2019) and Convolutional Neural Network (CNN) (Zeng *et al.* 2016), integrating them with the pre-trained k-mer embeddings. In addition to the k-mer embeddings learned by DNABERT, we compared these models with two other commonly used k-mer representations of dna2vec (Ng 2017) and one-hot encoding.

## 3 Results

### 3.1 Overlapping-consistent k-mer embeddings are learned by DNABERT for its token representation

To examine k-mer embeddings learned by DNABERT, we performed t-distributed Stochastic Neighbor Embedding (t-SNE) (Hinton and Roweis 2002) analysis using Scikit-learn (version 1.1.1) (Pedregosa *et al.* 2011). Given that a k-mer can be decomposed into nucleotides, we investigated k-mer embeddings in the context of their nucleotide composition. Our analysis focused on k-mer token embeddings and ignored other embeddings, such as token type and position embeddings. Token types, also known as segment IDs, are utilized to differentiate between two types of sequences in the input. In the pre-training task on nucleotide sequences, all token type embeddings were set to be the same. For the pre-trained model on the human reference genome, we directly utilized the models provided by DNABERT (accessible at https://github.com/jerryji1993/DNABERT, abbreviated as DNABERT_human). These models were trained on the human reference genome (GRCh38). To perform the t-SNE analysis, we extracted the weights of k-mer token embeddings from these provided models. With respect to the model pre-trained on randomly generated sequences (referred to as DNABERT_dataRand), we employed DNABERT with its default model hyperparameters for pre-training. We extracted the k-mer embeddings learned with randomly generated sequences from the trained model. To compare the learned k-mer embeddings with other widely used k-mer representations, we also visualized k-mer embeddings learned by dna2vec as well as one-hot encoding. Notably, neither of these two embeddings includes special tokens.

**Figure 3.**
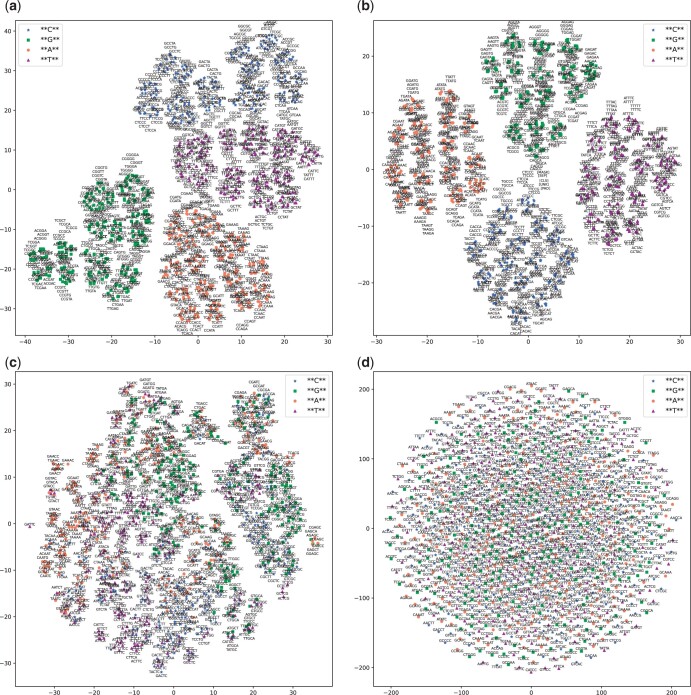
T-SNE plot of 5-mer embeddings generated by different methods (DNABERT_human, DNABERT_dataRand, dna2vec, and one-hot encoding). DNABERT_human refers to the DNABERT model pre-trained on the human reference genome. DNABERT_dataRand is the DNABERT model pre-trained on randomly generated sequences. Dna2vec is a k-mer embedding model based on word2vec trained on the human reference genome. One-hot encoding is a commonly used method for representing categorical data, such as nucleotides. (a) 5-mer embeddings learned by DNABERT pre-trained on human reference genome. (b) 5-mer embeddings learned by DNABERT pre-trained on randomly generated sequences. (c) 5-mer embeddings of dna2vec. (d) 5-mer embeddings of one-hot encoding.

Figure 3a and b illustrate the 5-mer embeddings learned by the DNABERT model with different pre-training datasets. From the figures, we have the following two observations. First, the special tokens appear distinctly separated from the other k-mer tokens within the visualized space. Second, among the k-mer tokens, there is a noticeable trend that k-mers sharing the same prefix or suffix nucleotide strings often cluster in close proximity. This is evident with k-mers like CTCCN, NGCTT, and NGAAT (“N” represents any nucleotide from A, T, C, G) as depicted in Fig. 3a and b. Such local clustering based on shared prefix and suffix strings subsequently contributes to the formation of larger aggregated clusters centered around a common nucleotide sequence, such as -GAT-. For visualization purposes, we color-coded each k-mer based on the nucleotide occupying its central position (--[A/T/G/C]--). This color-coding scheme facilitates the ease of visualization and analysis of the k-mer embeddings. In both Fig. 3a and b, four distinct clusters are readily observable, each corresponding to a nucleotide occupying the central position in a k-mer. Although randomly generated sequences lack inherent biological information, the contextual constraints of neighboring overlapping k-mers are retained in the random data. Compared with the DNABERT trained on the human genome, the k-mer embeddings from DNABERT pre-trained on randomly generated sequences are lined up more obviously. Here, we refer to the proximity of k-mer embeddings with similar prefixes or suffixes as “overlapping-consistent k-mer representation”. This pattern is evident in both the k-mer embeddings from models pre-trained on both real genomic sequences and random nucleotide sequences. The overlapping-consistent k-mer representation may be regarded as an inherent feature that DNABERT captures for the token representation during pre-training. Its species-independent character can be associated with the model’s generalization ability. For comparative analysis, we also visualized the two commonly used k-mer embeddings of dna2vec and one-hot encoding, as shown in Fig. 3c and d. In the dna2vec embedding, while not as pronounced as in Fig. 3a and b, the affinity of k-mers with identical prefixes or suffixes is still observable, exemplified by k-mers like NGAGC and GATGN in Fig. 3c. No larger clusters centered around the middle position are discernible in the t-SNE plot generated using dna2vec. As for the one-hot encoding, shown in Fig. 3d, the k-mers appear to be uniformly distributed.

We then evaluated DNABERT pre-trained models and assessed the efficacy of different k-mer embeddings in two downstream tasks of sequence-based functional prediction: TATA promoter prediction and TFBS prediction. We first evaluated the pre-trained DNABERT models in the context of the TATA promoter prediction task. Following the previous work (Oubounyt *et al.* 2019, Ji *et al.* 2021), we obtained the human and mouse positive promoter sequences from the EPDnew database (Dreos et al., 2017) . These positive sequences are from regions ranging from −249 to 50 bp surrounding the transcription start sites. The dataset comprises 29 598 positive human samples (TATA: 3065; non-TATA: 26 533) and 25 110 positive mouse samples (TATA: 3305; non-TATA: 21 805) with a length of 300 bp. Negative sequences were generated based on the characteristics of the positive samples. For samples containing the TATA motif, negative samples were selected from other genomic regions containing the TATA motif to preclude overly simplistic classifications based solely on the presence of the TATA motif. For the non-TATA sequences, the positive samples were generated by random substitution of subsequences. The complete dataset was divided into training, development, and test sets at an 80:10:10 ratio. Different pre-trained DNABERT models were fine-tuned utilizing the training and development sets, and subsequently evaluated on the test set.

**Figure 4.**
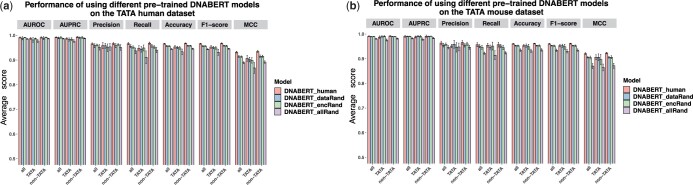
Performance comparison of different pre-trained DNABERT models fine-tuned on the TATA human (a) and mouse dataset (b). DNABERT_human is the provided model pre-trained on the human reference genome. DNABERT_dataRand is pre-trained on randomly generated sequences. DNABERT_encRand is based on DNABERT_human while the encoding module is randomly re-initialized. DNABERT_allRand is fine-tuning from scratch with all parameters randomly initialized. We carried out ten independent runs using different random seeds and reported average scores accompanied by their respective standard deviations.

The average performance of different DNABERT models, as determined through ten independent runs, is shown in Fig. 4. Besides DNABERT_human and DNABERT_dataRand, we compared with models where DNABERT_human had its encoding module or all modules randomly re-initialized. These models are named DNABERT_encRand and DNABERT_allRand, respectively. Detailed numerical results are available in Supplementary Table S1. When evaluated using single-threshold metrics (0.5) such as accuracy, F1-score (F1), and Matthews Correlation Coefficient (MCC), DNABERT_human outperforms both DNABERT_dataRand and DNABERT_encRand on the human and mouse datasets. However, when turning to performance metrics like AUROC and AUPRC, DNABERT_human, DNABERT_dataRand, and DNABERT_encRand exhibit comparable levels of performance. Among the four models assessed, DNABERT_allRand shows the lowest performance on both datasets. As illustrated in the figure, DNABERT_dataRand and DNABERT_encRand exhibit similar levels of performance. DNABERT_encRand retains the pre-trained weights of its embedding module from DNABERT_human but diverges in the weights associated with the encoding module. The observed performance disparity between DNABERT_encRand and DNABERT_human indicates the impact of encoding module, which takes the role of integrating information from lower-level encoding and embedding layers. DNABERT_dataRand was pre-trained on randomly generated data that lack inherent biological information. As a result, its encoding module cannot learn meaningful sequence patterns from such random data. On the other hand, the encoding module of DNABERT_encRand was randomly re-initialized. Regarding the functionality of identifying meaningful sequence patterns, the encoding modules of DNABERT_dataRand and DNABERT_encRand perform similarly. Besides that, the difference in the embedding module between DNABERT_dataRand and DNABERT_encRand does not lead to a significant performance difference in this downstream task.

### 3.2 Investigation of 1-mer DNABERT models

In addition to evaluating 5-mer DNABERT models, we assessed 1-mer DNABERT models pre-trained on both the human and randomly generated datasets. Distinct from the 5-mer models, the 1-mer models are not influenced by overlapping k-mer tokens. This feature allows the 1-mer model to focus on the second subtask of predicting masked nucleotides using contextual information from adjacent nucleotides. The 1-mer model failed to be pre-trained on the random data, but it converged successfully when pre-trained on the human data. There are significant differences in model weights and predicted nucleotide probabilities between models pre-trained on human data versus random data. We then evaluated the accuracy of masked tokens predicted by DNABERT_human using 100 000 samples randomly generated from human genome (15% masking rate) and acquired an accuracy of 0.5484. This result is consistent with a prior study (Liang *et al.* 2022), which indicates that the base prediction accuracy, given the surrounding context of 14 nucleotides, averages above 50%. These results from the 1-mer models provide additional support to the observation related to the DNABERT model pre-trained with overlapping k-mer tokens and the model learns the overlapping-consistent k-mer embeddings for its token representation. More detailed information on 1-mer models can be found in Section 5 of the Supplementary Material.

### 3.3 Evaluation of k-mer embeddings with a simpler model in the TATA promoter prediction task

**Figure 5.**
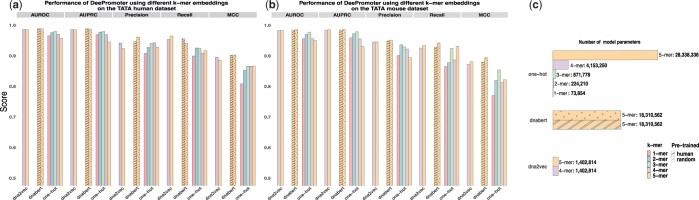
Performance of the DeePromoter model using different k-mer embeddings on the TATA human dataset (a) and mouse dataset (b). (c) The number of model parameters in DeePromoter using different k-mer embeddings.

Given the large number of model parameters within DNABERT, with encoding module accounting for ∼98.6% of the overall model parameters, we then explored the utilization of models with simpler encoding layers for downstream tasks. K-mer embeddings were investigated with a simplified model. We assessed four distinct k-mer embeddings and adopted the DeePromoter framework (Oubounyt *et al.* 2019) as the foundational architecture for the TATA promoter prediction task. Notably, compared with DNABERT, DeePromoter has a significantly reduced number of model parameters, as illustrated in Fig. 5c (or detailed in Supplementary Table S2). Despite its fewer parameters, DeePromoter has been reported to achieve a commendable performance. The k-mer embeddings under evaluation included one-hot encoding (ranging from 1-mer to 5-mer), dna2vec embeddings (for 4-mer and 5-mer), and 5-mer embeddings extracted from DNABERT, utilizing different pre-training datasets (human genome and random data). Unlike the default fine-tuning procedure of DNABERT, the weights of the embedding layer are no longer updated within the DeePromoter framework. The position and token type embeddings were not used in the simplified model. Our approach involved a comprehensive hyperparameter search, conducted on the human development set.

As illustrated in Fig. 5a and b, DeePromoter using k-mer embeddings from DNABERT has a better performance than those using the other two embedding methods of one-hot and dna2vec. With the one-hot encoding, the number of k-mers increases exponentially as *k* grows, resulting in the challenge of sparse high-dimensionality. The AUROC of the one-hot encoding model peaks at *k* = 3 and subsequently declines as *k* increases. The best performance of the one-hot k-mer model remains inferior to that of the models using dna2vec and DNABERT k-mer embeddings. Dna2vec employs the word2vec word embedding model, which is trained on a two-layer neural network, to learn k-mer embeddings. The provided dna2vec model supports variant k-mer lengths, with all the k-mer embeddings sharing a consistent dimension of 100. Although the dna2vec k-mer dimension is smaller than that of DNABERT (768), its performance in terms of AUROC, AUPRC, and MCC slightly lags behind models using DNABERT k-mer embeddings. For the DeePromoter model using k-mer embeddings derived from DNABERT, despite having significantly fewer model parameters than the full DNABERT model, the performances (both with human 5-mers and random 5-mers) are comparable to those achieved by DNABERT_dataRand and DNABERT_encRand on the human dataset (marginally lower on the mouse dataset). Interestingly, when employing k-mer embeddings of DNABERT pre-trained on random sequences, the DeePromoter model has an improved MCC result compared to the model using k-mer embeddings of DNABERT pre-trained on human data. This improvement is particularly notable in the mouse dataset. In conjunction with the observations from the t-SNE plot shown in Fig. 3, these results suggest that the overlapping-consistent k-mer embeddings learned by DNABERT stand out as a learning outcome for the token representation within the embedding module. Additionally, the k-mer embeddings learned by DNABERT provide a potent alternative to dna2vec and one-hot embeddings for encoding nucleotide sequences in deep learning frameworks.

### 3.4 Evaluation on TFBS prediction tasks

We extended the evaluation of related models on TFBS prediction tasks, including both motif discovery and motif occupancy identification (Zeng *et al.* 2016). Different from motif discovery that utilizes negative samples crafted from shuffled positive sequences preserving identical dinucleotide frequency, motif occupancy identification uses negative samples that are also from regions centering at a motif instance with matched size, GC, and motif strength. We evaluated a total of 690 motif discovery datasets and 422 motif occupancy datasets. These datasets contain input nucleotide sequences of 101 bp, which is shorter than the 300 bp input sequences used in the TATA promoter prediction task. Each dataset was randomly partitioned into 80% for training and 20% for testing. Within each training dataset, a subset of 12.5% was randomly selected to use as a development set. We utilized the CNN model (Zeng *et al.* 2016) as a simplified model for evaluating different k-mer embeddings. For comparative analysis, we also evaluated fine-tuned DNABERT models. We conducted model hyperparameter tuning using the default hyperparameters previously established for the CNN (Zeng *et al.* 2016) and DNABERT model as initial settings. Hyperparameter tuning for DNABERT was further optimized utilizing the development set of 20 randomly selected motif occupancy datasets. For each training set, models were trained for ten epochs and the model with the highest MCC score on the development set was saved. These stored models were used for predictions on the corresponding test sets.

**Figure 6.**
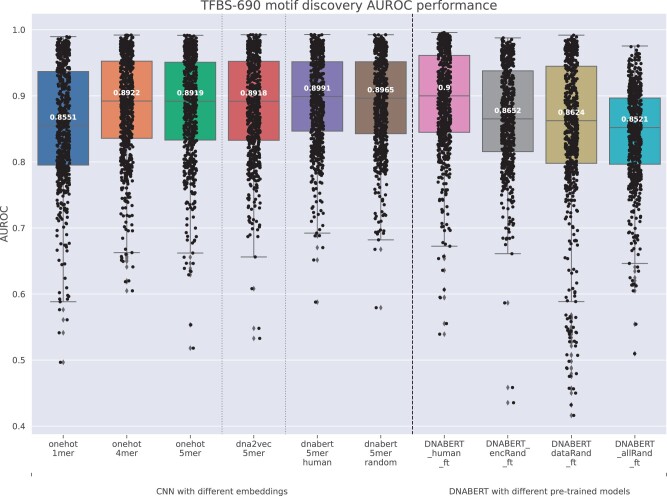
Boxplot of AUROC performance on 690 TFBS motif discovery datasets.

Figure 6 shows AUROC scores for CNN models using different k-mer embeddings and fine-tuned DNABERT models evaluated across 690 motif discovery datasets. We mainly focused on the median AUROC score of the 690 datasets as an overall assessment metric for different models. Additional metrics, including MCC, AUPRC, and F1, can be found in Section 4.1 of the Supplementary Material. For one-hot encoding, we evaluated k-mers of lengths 1, 4, and 5. The incorporation of one-hot encoding for 4-mer and 5-mer serves as a reference for comparative analysis against other learned k-mer embeddings, aligning their vocabulary sizes within a comparable scale range. The one-hot encoding of 1-mer yields a median AUROC score of 0.8551, in line with the previously reported result (Zeng *et al.* 2016). As the value of *k* increases to 4 and 5, the dimension of the input vector expands proportionally to 256 and 1024, resulting in improved median AUROC scores of 0.8929 and 0.8919 for 4-mer and 5-mer, respectively. Comparatively, the model using dna2vec embeddings performs similarly, achieving a median AUROC score of 0.8918, despite the dimensionality of the k-mer vector being restricted to 100. The median AUROC scores of the CNN model using DNABERT's pre-trained k-mer embeddings are also very close, which are 0.8991 and 0.8965 for k-mer embeddings derived from the model pre-trained on human data and random sequence data, respectively. The performance of fine-tuned DNABERT models is shown in the last four columns of Fig. 6. DNABERT_human_ft achieves the highest median AUROC score of 0.9. Both DNABERT_encRand_ft and DNABERT_dataRand_ft exhibit comparable performance levels, with scores of 0.8652 and 0.8624, respectively. These scores are lower than those achieved by the CNN models utilizing DNABERT's k-mer embeddings. DNABERT_allRand_ft without pre-training has the lowest median AUROC scores among all the compared models. These performance disparities among different pre-trained DNABERT models also demonstrate the impact of the encoding module in the downstream fine-tuning. Given the considerable variation in sample sizes across the 690 datasets, ranging from 139 to 143 425 (with a median of 21 929.5 for training data), the intricate encoding module with its large number of parameters poses challenges when relying solely on the fine-tuning dataset for training. This is particularly pronounced in the context of small-scale datasets. As a consequence, the necessity for adequate pre-training becomes apparent when employing a complex encoding module. In such a scenario, the pre-training process plays a crucial role in enabling the successful training of intricate models. On the other hand, our observations indicate that the performance disparity between CNN models utilizing k-mer embeddings and the DNABERT fine-tuning models is not notably significant for this particular task.

**Figure 7.**
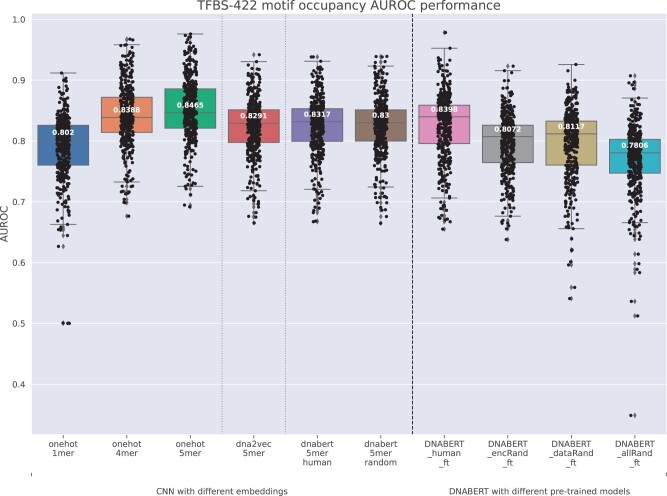
Boxplot of AUROC performances on 422 TFBS motif occupancy identification datasets.

Similarly, we conducted evaluations on 422 TFBS motif occupancy identification datasets, as illustrated in Fig. 7. Given that the motif occupancy identification is more challenging than the motif discovery task (Zeng *et al.* 2016), the median AUROC scores are generally lower, ranging from 0.78 to 0.8465, compared to the scores observed in the motif discovery task. Models employing the same type of k-mer embeddings exhibit a performance pattern that closely resembles what was observed in the motif discovery task. One different and interesting result is that the CNN model using 5-mer one-hot embedding achieves the highest median AUROC score of 0.8465, which is even slightly higher than the DNABERT_human_ft of 0.8398. Although it is difficult to perform strict comparisons between the two, this result indicates that a simpler neural network structure could be more suitable for tasks involving relatively small-scale datasets.

### 3.5 Acceleration of pre-training through model initializing using weights pre-trained on random data

Pre-training a BERT model is widely recognized as a process that is computationally intensive and time-consuming. Although a pre-trained model can be applied for cross-species applications, its efficacy may be confined to species with close phylogenetic relationships. For example, if a downstream application involves bacterial genomes, leveraging a model pre-trained on bacterial data may offer advantages over directly using a model pre-trained on human data. This consideration becomes pertinent when accounting for the role of encoding module. When there is a need to pre-train a new model, leveraging initial model weights from a model pre-trained on random data can accelerate the pre-training process. This becomes particularly beneficial once overlapping-consistent k-mer embeddings have been learned.

**Figure 8.**
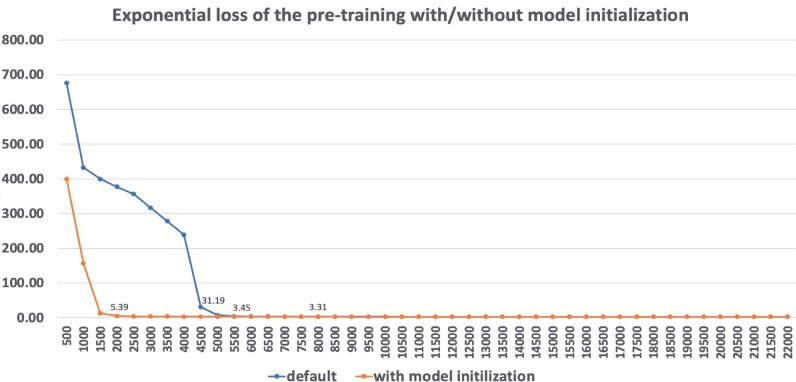
Exponential loss curve on the development set of the pre-training with and without model initialization. The development set is randomly sampled 20% of the total training dataset of the human reference genome. Applying model initialization using the weights of the model pre-trained on random data demonstrates efficacy in reducing the number of steps needed for convergence.

Figure 8 illustrates the exponential loss curve on the development set during pre-training with and without model initialization on the human reference genome. With the model initialization, the pre-training process approaches convergence after ∼1500 steps. In contrast, pre-training without the model initialization begins to show signs of convergence after about 5500 steps. Given that pre-training on random sequence data is non-species-specific, its utilization introduces no additional bias into the newly pre-trained model.

## 4 Discussion

In this work, we conducted a comprehensive analysis of the BERT model pre-trained on nucleotide sequences. Different from conventional analyses, we proposed to use a non-standard pre-training approach that incorporated randomness at both the data and model levels. We subsequently contrasted these variant pre-trained models (or modules) with their standard pre-trained counterparts, drawing a parallel to the “knockout” versus “wild type” comparisons commonly made in wet-lab experiments. Our experiments and analyses revealed that when pre-training on the task of predicting contiguous masked overlapping k-mers, one key feature learned for the token representation is the overlapping-consistent k-mer embeddings. These embeddings effectively capture the similarity structure of overlapping k-mers and can be independently leveraged for various downstream tasks. To further refine the pre-training task for nucleotide sequences, exploring tokenization methods informed by biological insights and designing pre-training tasks of heightened biological significance would be beneficial.

For protein sequences, previous work (Yang *et al.* 2018) has demonstrated that randomizing the UniPort sequences improves downstream performance for some tasks, such as cytochrome P450 thermostability. This improvement was hypothesized to be related to a regularizing effect on the embedding model. Specifically, the randomization deters the embedding model from overfitting to a particular subset of protein sequences in the pre-training dataset. In this study, we used randomization from a different motivation for analyzing the DNABERT model for nucleotide sequences. Another notable difference between the two works is the unit of biological sequence representation. DNABERT generally uses overlapping k-mers for representing nucleotide sequences, whereas protein sequences utilize non-overlapping amino acids as their foundational unit. Our assessment across two downstream tasks showed that k-mer embeddings learned from randomized nucleotide sequences can also be helpful in downstream tasks in the pre-training and fine-tuning framework. These embeddings learned from random data can provide close performance comparable to the overlapping k-mer embeddings learned on real genomic data. Furthermore, k-mer embeddings trained on the random data can provide an unbiased option of overlapping k-mer representation for downstream tasks.

One commonly used approach to highlight the impact of pre-training is the practice of training models from scratch. This method skips the pre-training phase, moving directly to the fine-tuning process. Typically, such scratch-training results in a lower performance (Ji *et al.* 2021), as models with a large number of parameters are difficult to be trained only using a limited fine-tuning dataset. In this study, we introduced two additional approaches to illustrate the impact of pre-training. Besides introducing randomness into the encoding module, we also conducted pre-training on totally random sequences. Compared with introducing randomness into the encoding module, using the pre-trained model on random sequences achieves relatively close results in the TATA promoter prediction and TFBS prediction tasks. The pre-trained model on randomized data can provide a pivot to investigate biologically insignificant perturbations of input nucleotide sequences.

## 5 Conclusion

In this study, we focused on nucleotide sequences and analyzed the learning outcomes of a typical BERT model learned through pre-training. We applied a non-standard pre-training methodology to scrutinize different modules by incorporating randomness at both the data and model levels. We demonstrated that the overlapping-consistent embedding of k-mers is the outcome that the DNABERT model learns for its token representation. We assessed the efficacy of utilizing different pre-trained k-mer embeddings and compared them with other commonly used k-mer representations in downstream tasks related to sequence-based functional prediction. Additionally, we demonstrated that utilizing a model pre-trained on random sequences can accelerate the pre-training process.

## Supplementary Material

btad617_Supplementary_Data
