# Supplementary material for: Investigation of the BERT model on nucleotide sequences with non-standard pre-training and evaluation of different k-mer embeddings
Source: Bioinformatics. 2023 Oct 10;39(10):btad617. doi: 10.1093/bioinformatics/btad617 (PMC10612406; doi:10.1093/bioinformatics/btad617)
Supplement: btad617_Supplementary_Data [file btad617_supplementary_data.pdf]

Supplementary materials

1.Data

1.1 Random sequence generation and pre-training data preparation

We used python (version 3.8.10) and specifically utilized the '***random.choice'*** function to repeatedly to generate random sequences. The default random seed for our experiment was set to ***123***. In total, we generated a random sequence comprising 3 billion nucleotides. Subsequently, we prepared the pre-training data for random sequences using '***process_pretrain_data.py'*** provided by DNABERT (Ji et al., 2021).

2. Models

2.1 DNABERT fine-tuning models

We used the pre-trained models provided by DNABERT (Ji et al., 2021).
https://drive.google.com/file/d/1KMqgXYCzrrYD1qxdyNWnmUYPtrhQqRBM/view

For the fine-tuning, based on the reported hyperparameters of DNABERT, we experimented with learning rate of [1e-4, 2e-4 and 5e-5] on the development sets (TATA_human and 20 randomly selected TFBS-422). We employed the lr that yielded the highest MCC performance on the corresponding development set. The fine-tuning learning rate of DNABERT, DNABERT_dataRand, DNABERT_encRand and DNABERT_allRand are 1e-4, 5e-5, 5e-5, and 5e-5, respectively. These lrs were used for the subsequent experiments.

TATA-task fine-tuning

Pre-trained model	learning rate	batch size	dropout	warmup	num epochs	weight decay
**DNABERT_human**	1e-4	64	0.1	0.06	20	0.1
**DNABERT_dataRand**	5e-5	64	0.1	0.06	20	0.1
**DNABERT_encRand**	5e-5	64	0.1	0.06	20	0.1
**DNABERT_allRand**	5e-5	64	0.1	0.06	20	0.1

TFBS-task fine-tuning

Pre-trained_model	learning rate	batch size	dropout	warmup	num epochs	weight decay
**DNABERT_human**	1e-4	64	0.1	0.1	10	0.01
**DNABERT_dataRand**	5e-5	64	0.1	0.1	10	0.01
**DNABERT_encRand**	5e-5	64	0.1	0.1	10	0.01
**DNABERT_allRand**	5e-5	64	0.1	0.1	10	0.01

Maximum_seq_len = input_seq_len + 2 – k_mer_length +1, where the value 2 accounts for the number of special tokens.

2.2 DeePromoter with different k-mers

2.2.1 Hyperparameters

KMER=5
SPIECE="mouse/human"

MODEL="deepPromoterNet"
EMBEDDING="onehot/dna2vec/dnabert"
KERNEL="5,5,5"
LR=1e-4
EPOCH=20
DROPOUT=0.1
BS=64

2.2.2 Model structure

```
deepPromoterNet(
  (cnn_diff_kernel): ModuleList(
    (0): Sequential(
      (0): Conv1d(16, 16, kernel_size=(5,), stride=(1,), padding=same)
      (1): ReLU(inplace=True)
      (2): MaxPool1d(kernel_size=6, stride=6, padding=0, dilation=1, ceil_mode=False)
      (3): Dropout(p=0.1, inplace=False)
    )
    (1): Sequential(
      (0): Conv1d(16, 16, kernel_size=(5,), stride=(1,), padding=same)
      (1): ReLU(inplace=True)
      (2): MaxPool1d(kernel_size=6, stride=6, padding=0, dilation=1, ceil_mode=False)
      (3): Dropout(p=0.1, inplace=False)
    )
    (2): Sequential(
      (0): Conv1d(16, 16, kernel_size=(5,), stride=(1,), padding=same)
      (1): ReLU(inplace=True)
      (2): MaxPool1d(kernel_size=6, stride=6, padding=0, dilation=1, ceil_mode=False)
      (3): Dropout(p=0.1, inplace=False)
    )
  )
  (biLSTM): LSTM(49, 32, batch_first=True, bidirectional=True)
  (linear): Linear(in_features=64, out_features=32, bias=True)
  (flatten): Flatten(start_dim=1, end_dim=-1)
  (fc): Sequential(
    (0): Linear(in_features=1536, out_features=128, bias=True)
    (1): ReLU()
    (2): Dropout(p=0.1, inplace=False)
    (3): Linear(in_features=128, out_features=2, bias=True)
    (4): Softmax(dim=1)
  )
)
```

2.3 CNN (Zeng et al.,) with different k-mers

2.3.1 Hyperparameters

MODEL="zeng_CNN"
KERNEL=24
EMBEDDING="onehot/dna2vec/dnabert"
LR=0.001
EPOCH=10
BS=64
dropout=0.1

2.3.2 Model structure:

```
zeng_CNN(
  (cnn_1m): Sequential(
    (0): Conv1d(voc_size, 128, kernel_size=(24,), stride=(1,), padding=same)
    (1): ReLU(inplace=True)
  )
  (fc): Sequential(
    (0): Linear(in_features=128, out_features=32, bias=True)
    (1): ReLU()
    (2): Dropout(p=0.1, inplace=False)
    (3): Linear(in_features=32, out_features=2, bias=True)
    (4): Softmax(dim=1)
  )
)
```

2.4 GPU usage

Usage	GPU card	Number
**DeePromoter + different embeddings**	Nvidia A6000	1
**CNN + different embeddings**	Nvidia A6000	1
**DNABERT pre-training**	Nvidia A6000	2
**DNABERT fine-tuning**	Nvidia V100	2 (TATA) / 1 (TFBS)

For reproducibility, the default random seed is set to be **123**.

3. Supplemental Figures and Tables

To improve visibility, enlarged figures are incorporated in this section.

3.1 Supplemental Figures

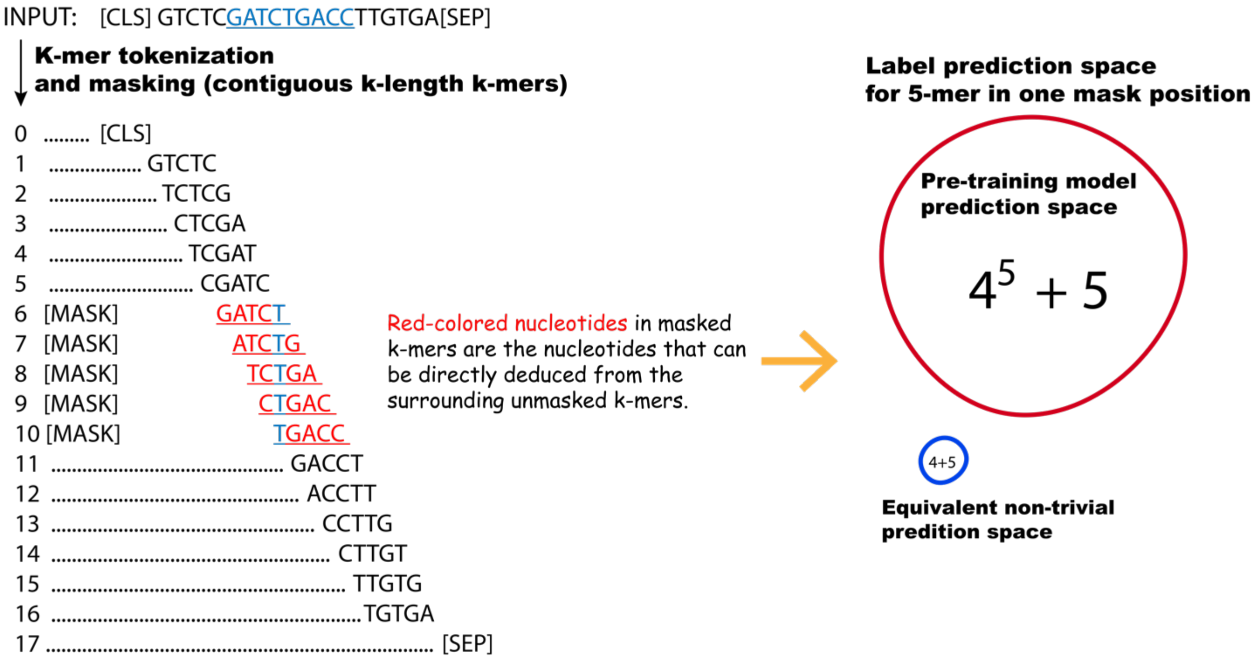

Figure S1 K-mer token masking strategy used in DNABERT. A nucleotide sequence is first converted into an overlapping k-mer sequence, with special tokens inserted at both ends of the sequence. For pre-training, partial tokens are masked to train the model for their prediction. To preclude the trivial inference of a masked token from its immediately adjacent k-mers, DNABERT masks *k* contiguous k-mers. Compared with the prediction space of the non-trivially inferable nucleotides (those that cannot be directly deduced from adjacent k-mers), the original prediction space is appreciably larger.

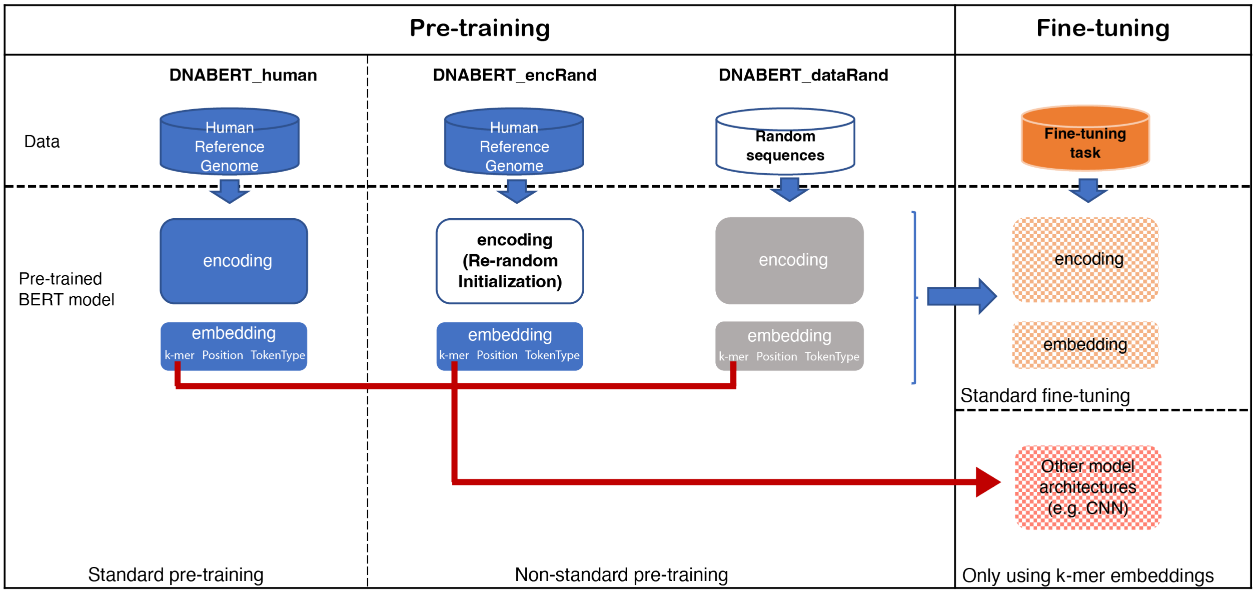

Figure S2 Model decomposition and analysis strategy for the DNABERT model. The pre-trained model is decomposed into embedding and encoding modules. To investigate the BERT model, we carried out non-standard pre-training that incorporates randomness at both the data and model levels. At the data level, we generated random nucleotide sequences for ~3 billion bases to compare with the DNABERT pre-trained on the human reference genome. At the model level, we incorporated randomness into encoding module through the re-initialization of corresponding model weights. In the fine-tuning stage, in addition to the standard fine-tuning, we also assessed simpler neural network structures that solely utilize the learned k-mer embeddings.

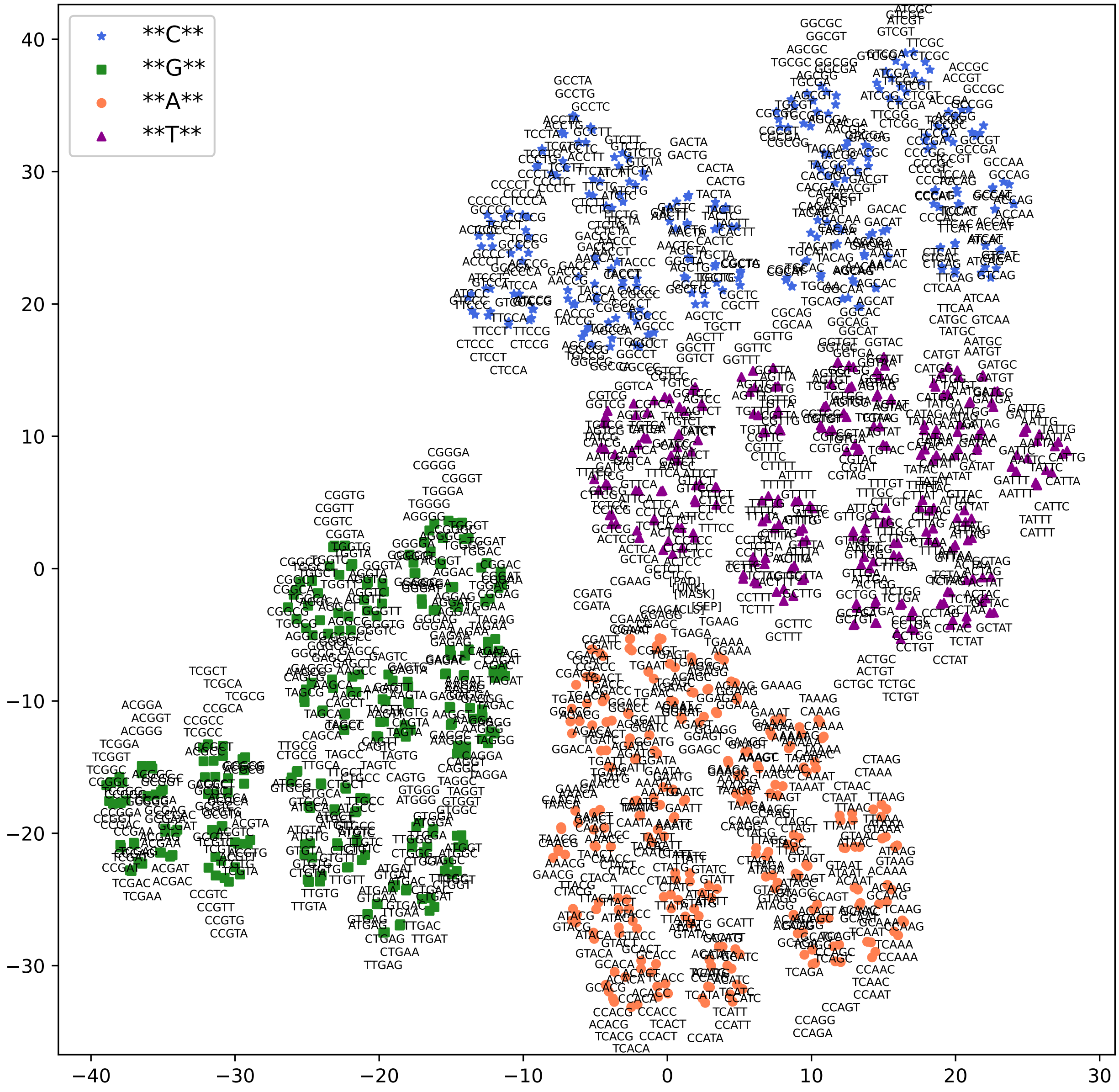

Figure S3 T-SNE plot of the 5-mer embedding of DNABERT (provided by Ji et al., 2021) pre-trained on human genome data.

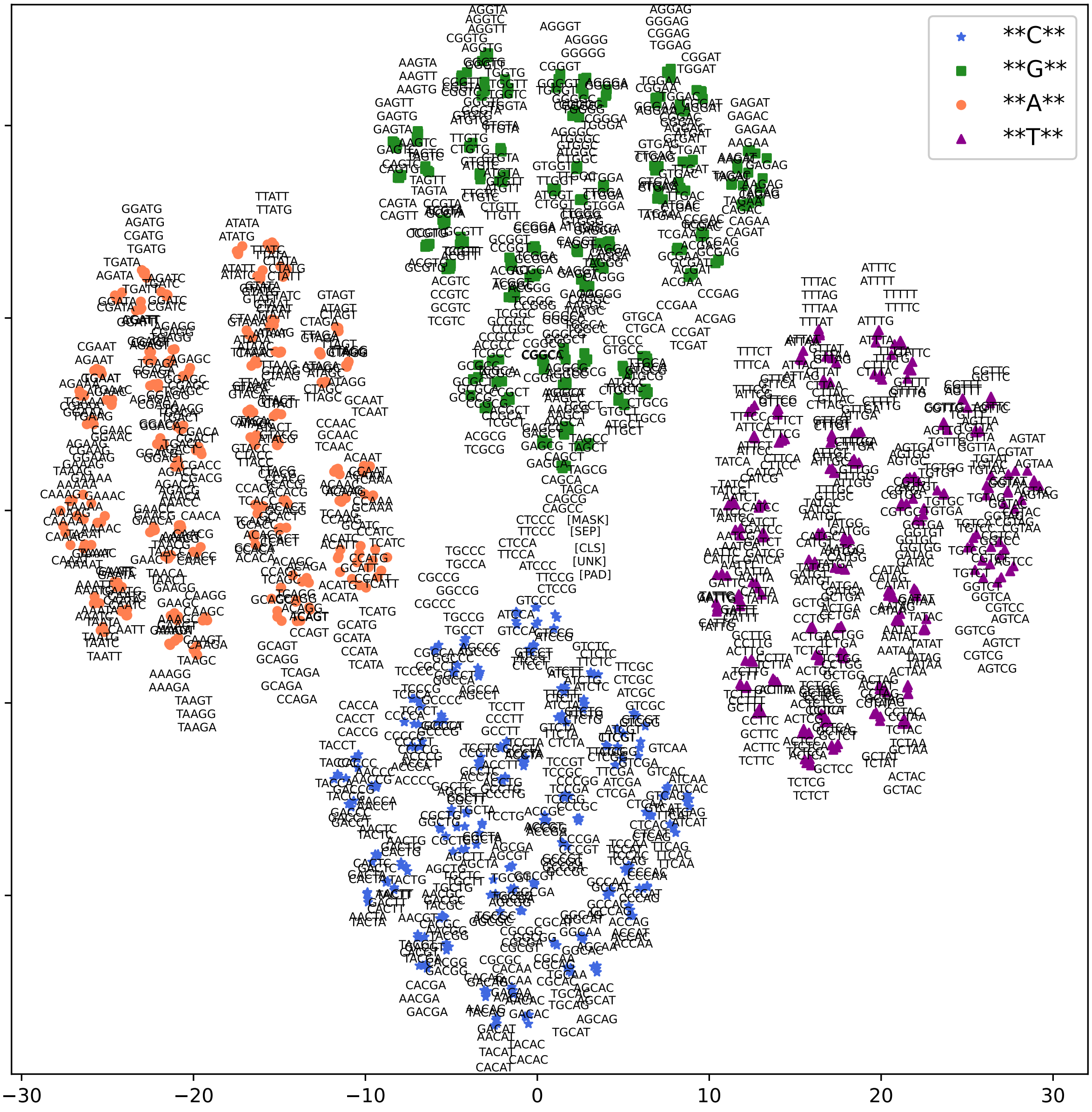

Figure S4 T-SNE plot of the 5-mer embedding of DNABERT pre-trained on completely random nucleotide sequences.

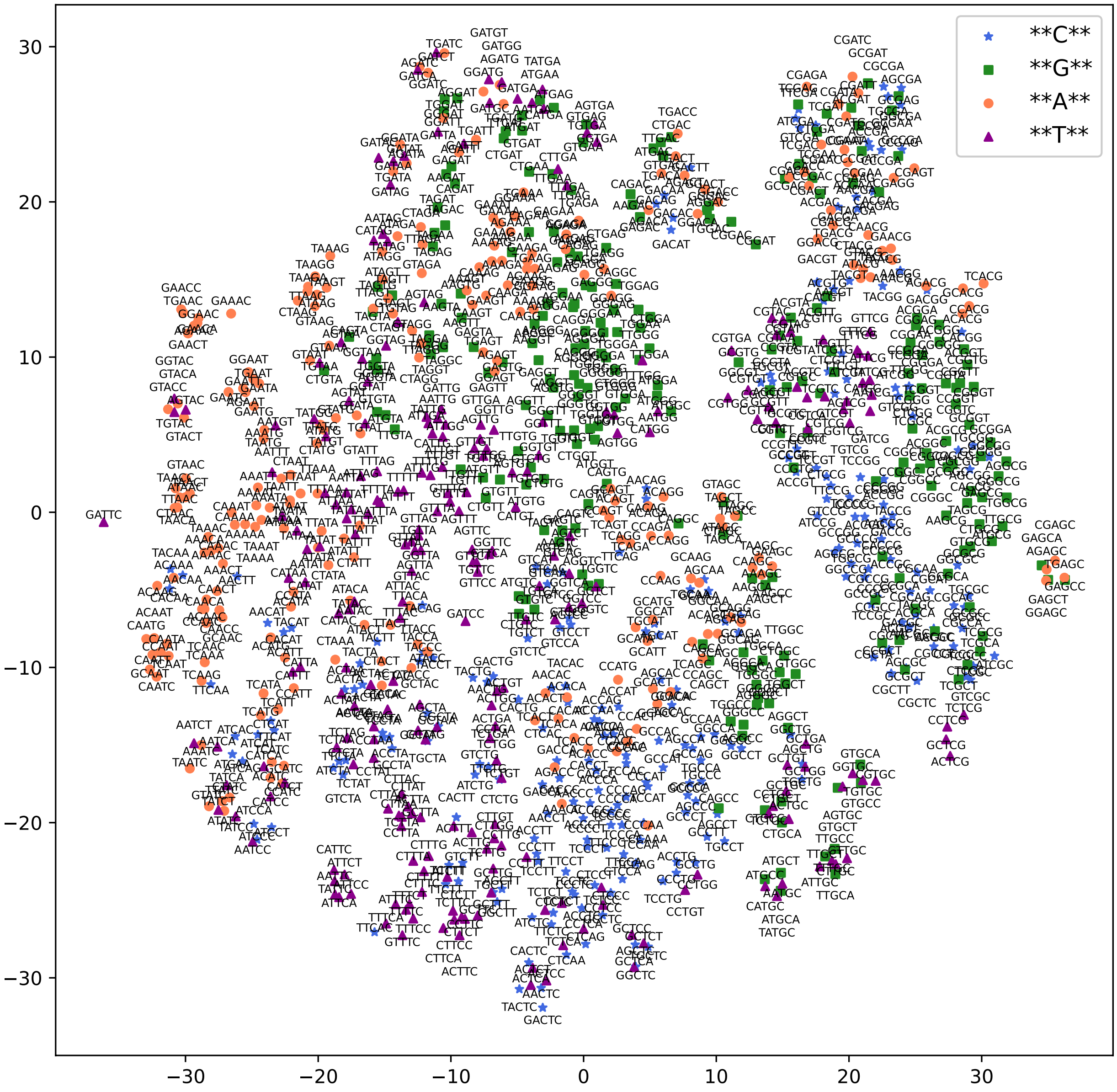

Figure S5 T-SNE plot of the 5-mer dna2vec embedding (provided by Ng. et al., 2017).

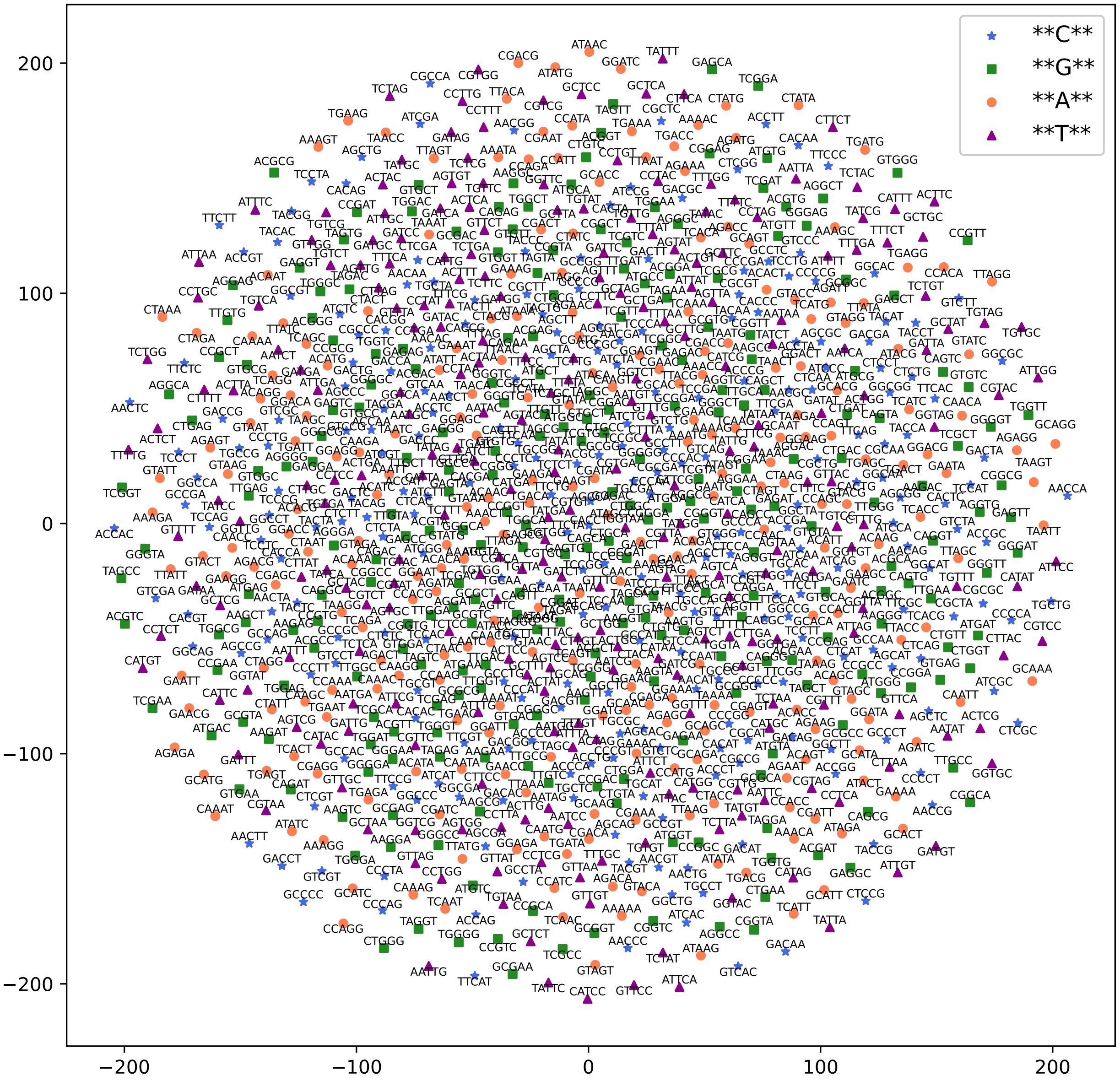

Figure S6 T-SNE plot of the 5-mer one-hot encoding.

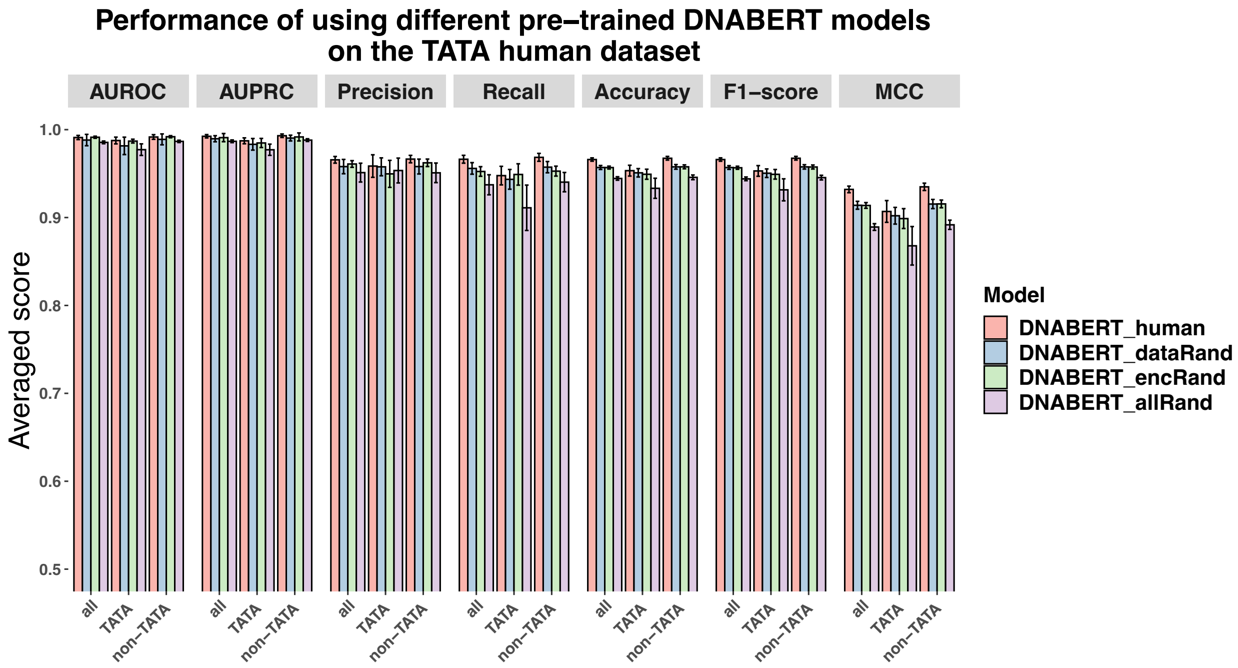

Figure S7 Performance of different pre-trained DNABERT models on the TATA human dataset.

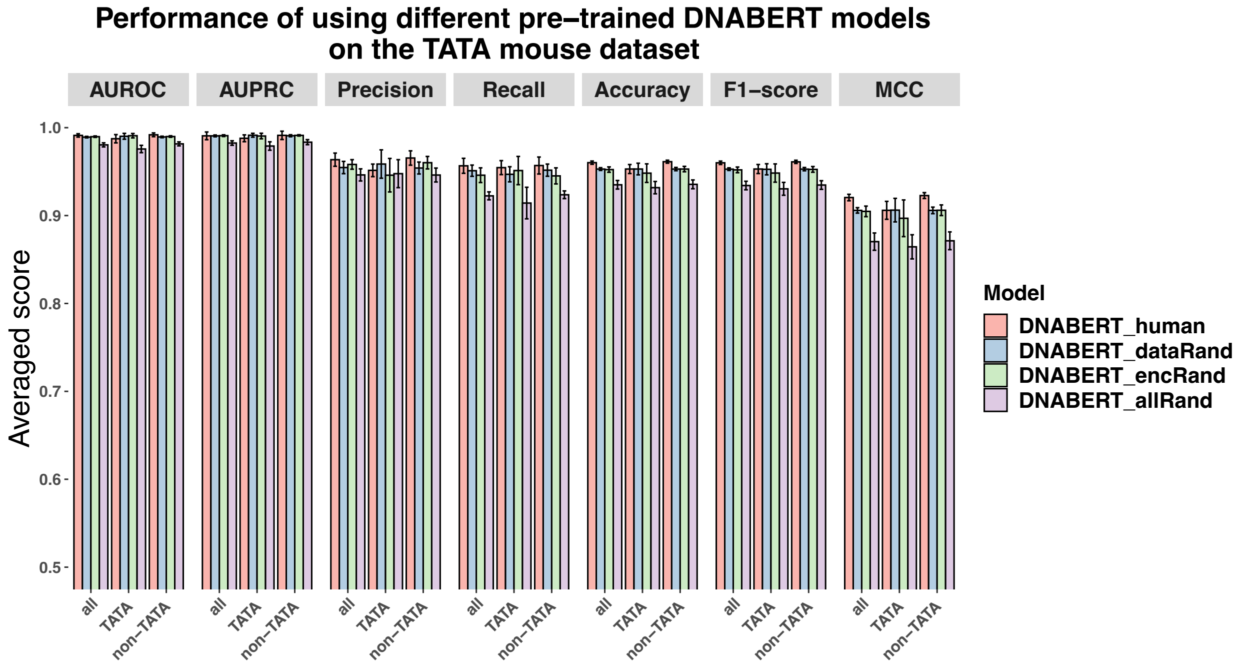

Figure S8 Performance of different pre-trained DNABERT models on the TATA mouse dataset.

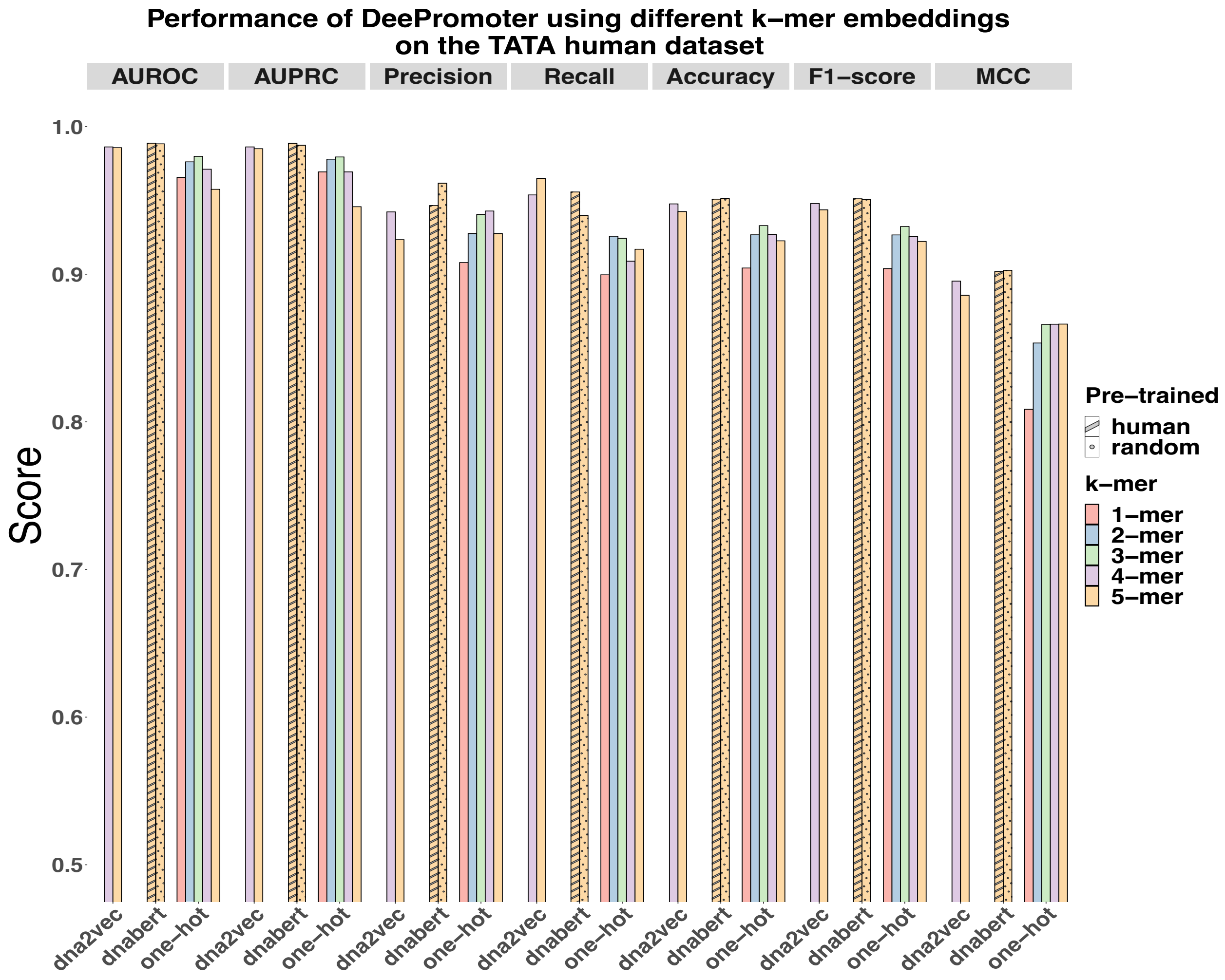

Figure S9 Performance of DeePromoter model using different k-mer embeddings on the TATA human dataset.

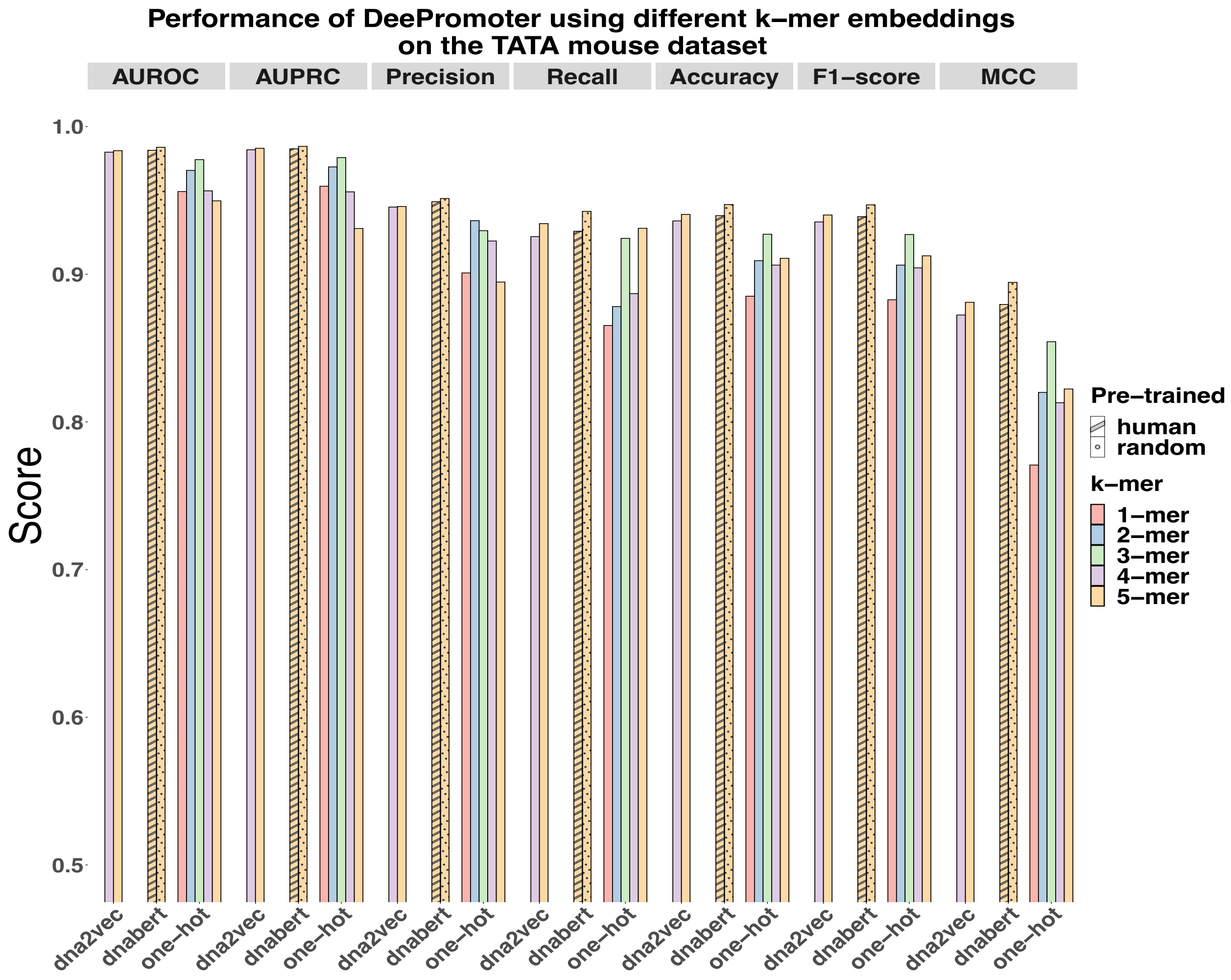

Figure S10 Performance of DeePromoter model using different k-mer embeddings on the TATA mouse dataset.

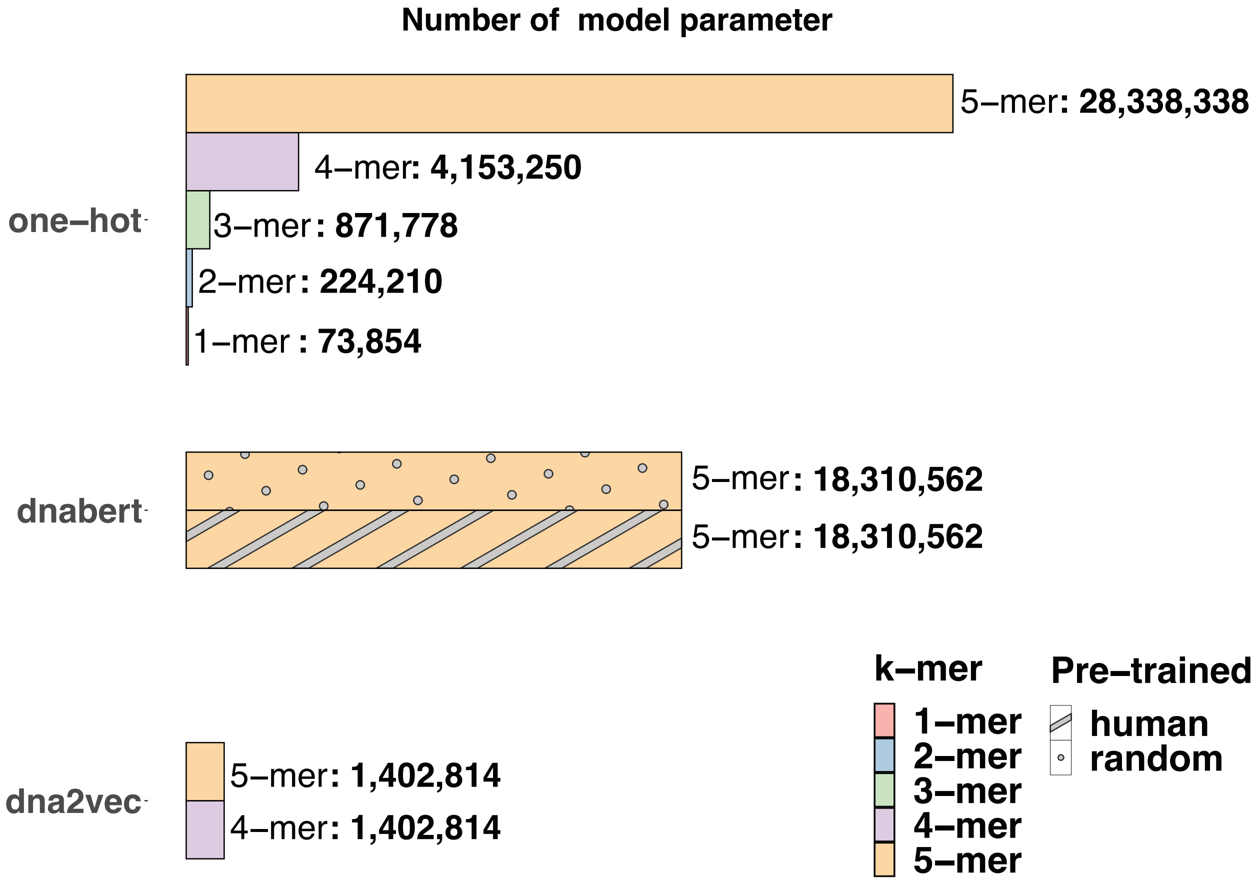

Figure S11 Number of model parameters in DeePromoter using different k-mer embeddings.

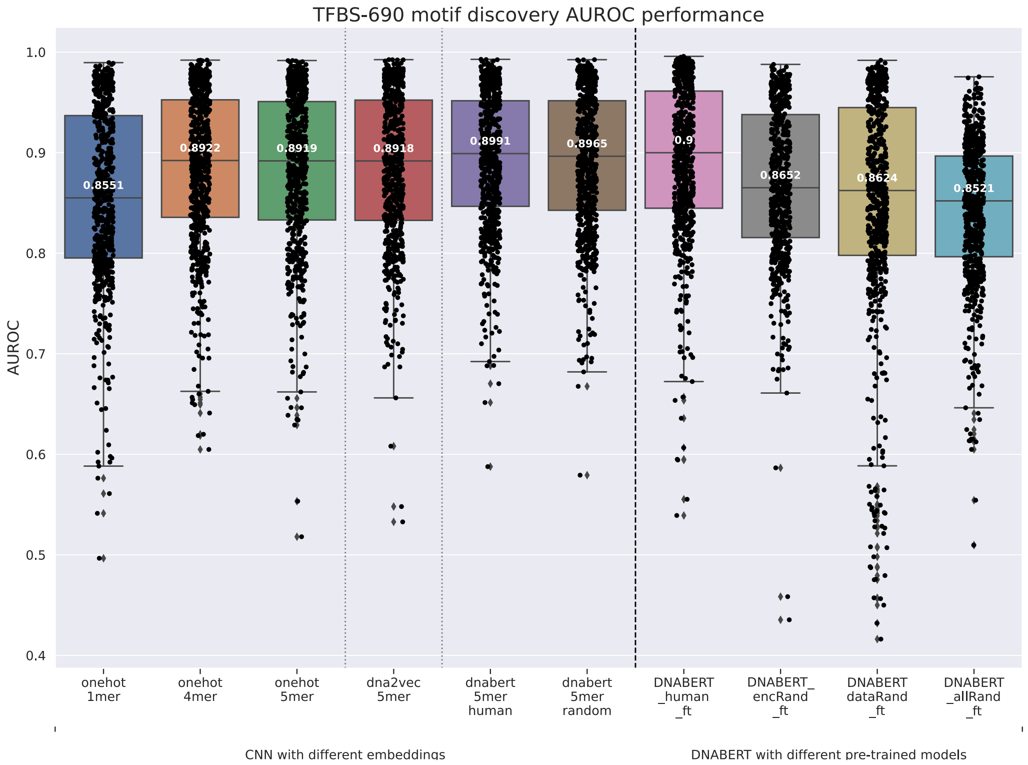

Figure S12 Boxplot of AUROC performance on 690 TFBS motif discovery datasets.

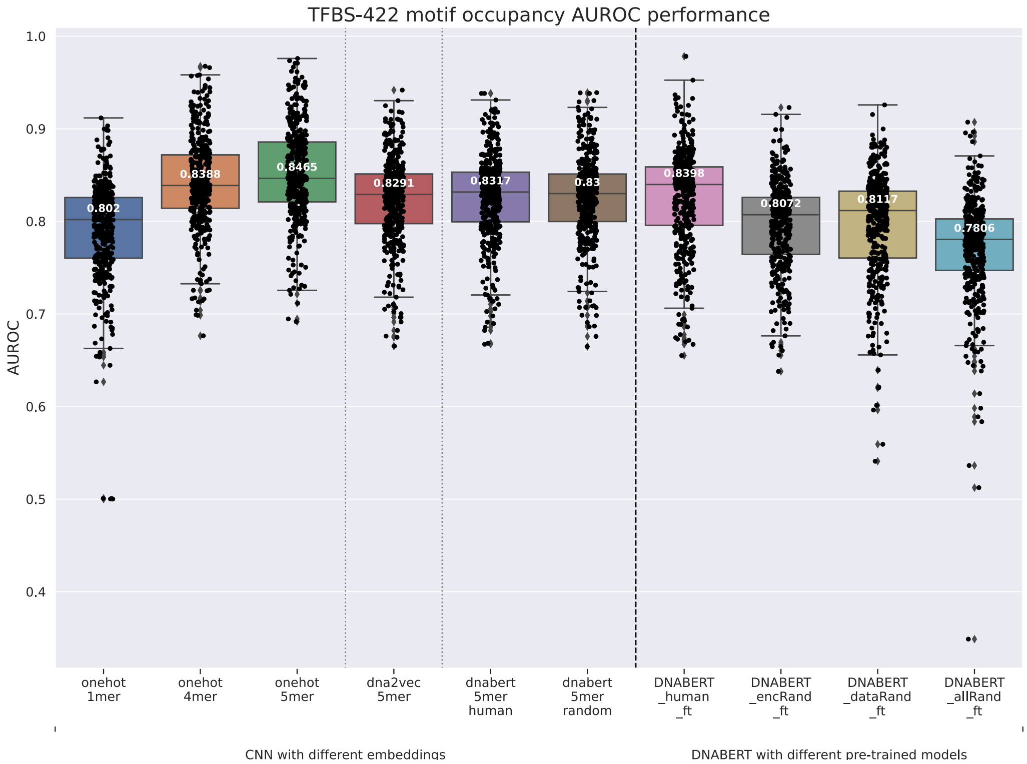

Figure S13 Boxplot of AUROC performances on 422 TFBS motif occupancy identification datasets.

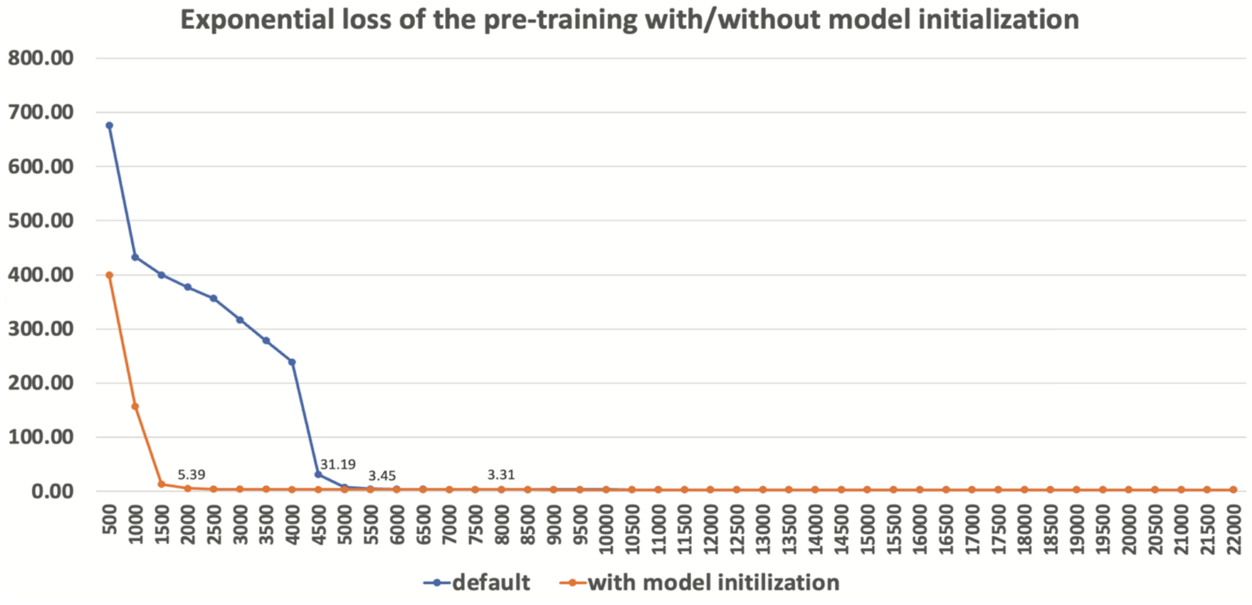

Figure S14 Exponential loss curve on the development set of the pre-training with and without model initialization. The development set is randomly sampled 20% of the total training dataset of the human reference genome. Applying model initialization using the weights of the model pre-trained on random data demonstrates efficacy in reducing the number of steps needed for convergence.

3.2 Tables

Human dataset

Pre-trained model	Test data category	AUROC	AUPRC	Precision	Recall	Accuracy	F1-score	MCC
DNABERT_human	all	0.9911	0.9925	0.9657	0.9663	0.9660	0.9660	0.9320
	TATA	0.9876	0.9873	0.9586	0.9477	0.9533	0.9530	0.9069
	non-TATA	0.9916	0.9931	0.9665	0.9685	0.9675	0.9675	0.9349
DNABERT_dataRand	all	0.9882	0.9897	0.9580	0.9559	0.9569	0.9569	0.9140
	TATA	0.9815	0.9833	0.9577	0.9435	0.9509	0.9504	0.9020
	non-TATA	0.9889	0.9904	0.9580	0.9573	0.9576	0.9576	0.9154
DNABERT_encRand	all	0.9914	0.9909	0.9609	0.9524	0.9568	0.9566	0.9138
	TATA	0.9870	0.9848	0.9497	0.9490	0.9493	0.9492	0.8988
	non-TATA	0.9920	0.9917	0.9623	0.9528	0.9577	0.9575	0.9155
DNABERT_allRand	all	0.9855	0.9868	0.9511	0.9373	0.9445	0.9440	0.8892
	TATA	0.9772	0.9771	0.9535	0.9111	0.9333	0.9315	0.8679
	non-TATA	0.9867	0.9881	0.9509	0.9403	0.9458	0.9454	0.8918

Mouse dataset

Pre-trained model	Test data	AUROC	AUPRC	Precision	Recall	Accuracy	F1-score	MCC
DNABERT_human	all	0.9912	0.9907	0.9636	0.9566	0.9602	0.9600	0.9205
	TATA	0.9874	0.9879	0.9514	0.9545	0.9530	0.9530	0.9060
	non-TATA	0.9919	0.9913	0.9655	0.9569	0.9613	0.9611	0.9228
DNABERT_dataRand	all	0.9893	0.9907	0.9547	0.9510	0.9529	0.9528	0.9059
	TATA	0.9902	0.9914	0.9586	0.9470	0.9530	0.9526	0.9062
	non-TATA	0.9894	0.9908	0.9541	0.9517	0.9529	0.9528	0.9059
DNABERT_encRand	all	0.9897	0.9909	0.9582	0.9459	0.9523	0.9520	0.9048
	TATA	0.9909	0.9905	0.9459	0.9512	0.9483	0.9484	0.8969
	non-TATA	0.9899	0.9912	0.9602	0.9451	0.9529	0.9525	0.9061
DNABERT_allRand	all	0.9804	0.9825	0.9462	0.9224	0.9350	0.9342	0.8703
	TATA	0.9757	0.9791	0.9477	0.9142	0.9318	0.9304	0.8644
	non-TATA	0.9816	0.9835	0.9461	0.9237	0.9355	0.9347	0.8713

Table S1 Comparison of using different pre-trained DNABERT models on TATA task. Averaged values of ten repeats of randomly generated seeds are reported. The ten random seeds are 86, 141, 118, 275, 634, 755, 4, 988, 826 and 478. All models were fine-tuned on the TATA training set and evaluated on the test set. DNABERT_human is the provided model pre-trained on human reference genome. DNABERT_dataRand is pre-trained on totally randomly generated sequences.DNABERT_encRand is based on DNABERT_human while the encoding layers are randomly re-initialized. DNABERT_allRand is the random weight model without pre-training.

Human dataset

Embedding	k-mer	Voc size	Model parameter	AUROC	AUPRC	Precision	Recall	Accuracy	F1-score	MCC
one-hot	1-mer	4	73854	0.9655	0.9693	0.9079	0.8996	0.9042	0.9038	0.8085
	2-mer	16	224210	0.9761	0.9779	0.9275	0.9257	0.9267	0.9266	0.8534
	3-mer	64	871778	0.9798	0.9794	0.9405	0.9243	0.9329	0.9323	0.8660
	4-mer	256	4153250	0.9711	0.9693	0.9428	0.9088	0.9269	0.9255	0.8543
	5-mer	1024	28338338	0.9575	0.9457	0.9275	0.9169	0.9226	0.9222	0.8453
dna2vec	4-mer	100	1402814	0.9862	0.9862	0.9422	0.9537	0.9476	0.9479	0.8953
	5-mer	100	1402814	0.9857	0.9850	0.9234	0.9649	0.9424	0.9436	0.8857
dnabert	human 5-mer	768	18310562	**0.9887**	0.9886	0.9465	0.9557	0.9508	0.9511	0.9017
	random 5-mer	768	18310562	0.9883	0.9873	0.9616	0.9398	0.9512	0.9506	**0.9026**

Mouse dataset

Embedding	k-mer	Voc size	Model parameter	AUROC	AUPRC	Precision	Recall	Accuracy	F1-score	MCC
one-hot	1-mer	4	73854	0.9560	0.9596	0.9009	0.8653	0.8851	0.8827	0.7708
	2-mer	16	224210	0.9703	0.9727	0.9363	0.8781	0.9092	0.9062	0.8200
	3-mer	64	871778	0.9776	0.9790	0.9295	0.9243	0.9271	0.9269	0.8543
	4-mer	256	4153250	0.9565	0.9558	0.9225	0.8869	0.9062	0.9043	0.8130
	5-mer	1024	28338338	0.9497	0.9310	0.8947	0.9311	0.9108	0.9125	0.8223
dna2vec	4-mer	100	1402814	0.9827	0.9843	0.9455	0.9255	0.9361	0.9354	0.8724
	5-mer	100	1402814	0.9837	0.9853	0.9459	0.9343	0.9405	0.9401	0.8810
dnabert	human 5-mer	768	18310562	0.9839	0.9849	0.9491	0.9291	0.9397	0.9390	0.8795
	random 5-mer	768	18310562	**0.9860**	0.9866	0.9513	0.9426	0.9472	0.9470	**0.8945**

Table S2 Comparison of different k-mer embeddings used by the DeePromoter model (Default seed=123). The model parameters were selected according to the best MCC performance on the development set. The best AUROC and MCC scores are shown in bold.

4. Supplemental Experiments

4.1 Other evaluation metrics on TBFS tasks

The performance metrics (AUROC, AUPRC, F1-score, and MCC) were calculated for each ChIP-seq dataset. Besides the AUROC shown in the main text, the overall boxplots of other evaluation metrics (AUPRC, F1-score, and MCC) are provided in this section.

4.1.1 TBFS motif discovery (690)

AUPRC

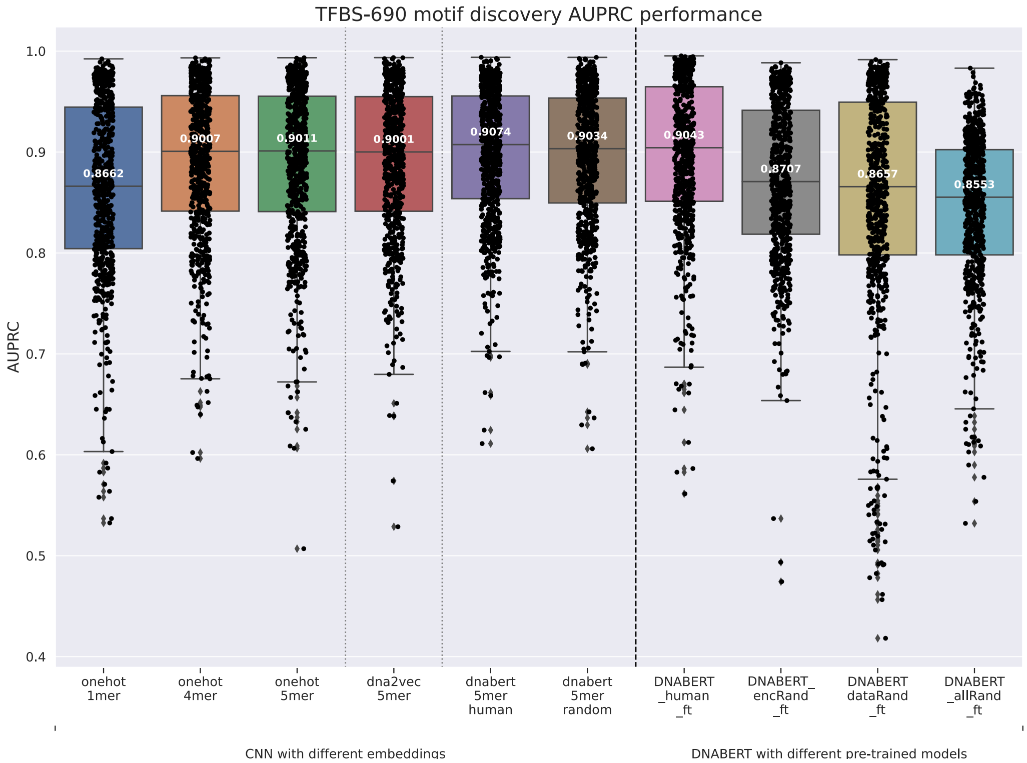

F1-score

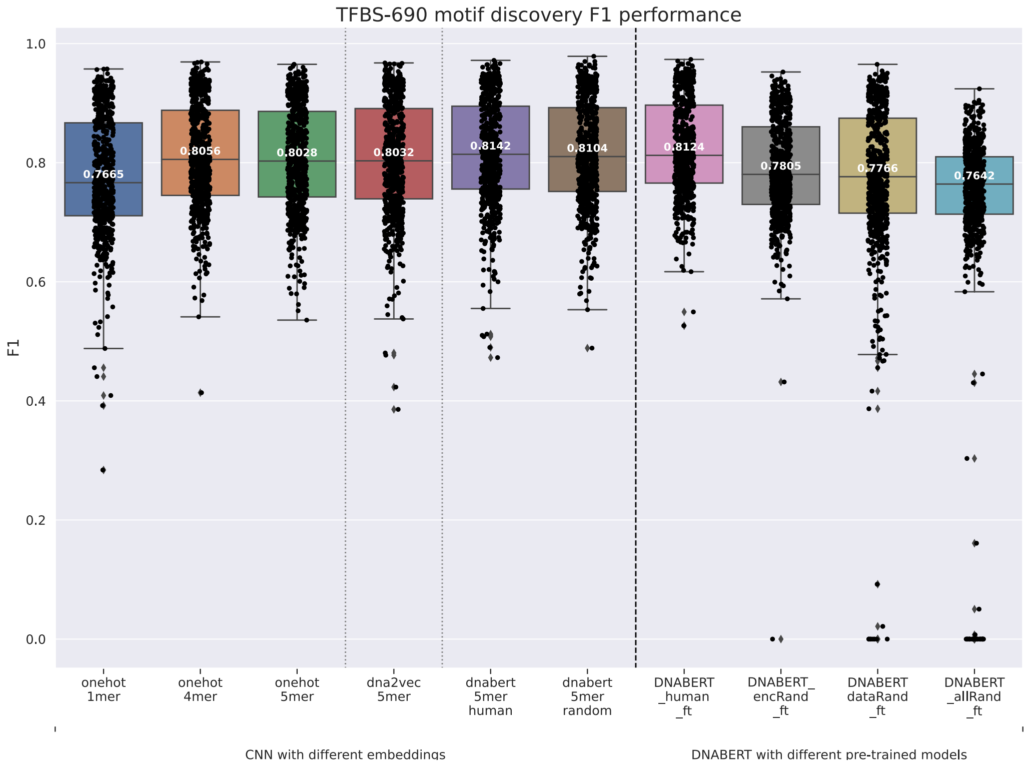

TFBS-690 motif discovery F1 performance
F1
1.0
0.8
0.6
0.4
0.2
0.0
0.7665
0.8056
0.8028
0.8032
0.8142
0.8104
0.8124
0.7805
0.7766
0.7642
onehot 1mer
onehot 4mer
onehot 5mer
dna2vec 5mer
dnabert 5mer human
dnabert 5mer random
DNABERT _human _ft
DNABERT_ encRand _ft
DNABERT dataRand _ft
DNABERT _allRand _ft
CNN with different embeddings
DNABERT with different pre-trained models

MCC

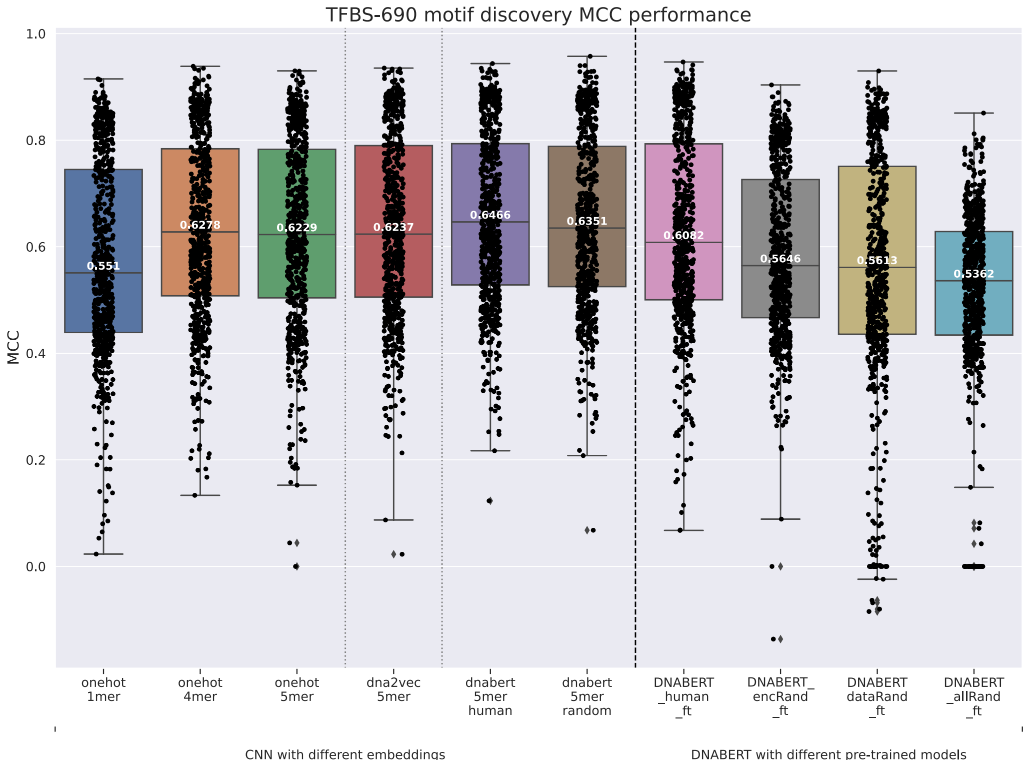

TFBS-690 motif discovery MCC performance
MCC
1.0
0.8
0.6
0.4
0.2
0.0
0.551
0.6278
0.6229
0.6237
0.6466
0.6351
0.6082
0.5646
0.5613
0.5362
onehot 1mer
onehot 4mer
onehot 5mer
dna2vec 5mer
dnabert 5mer human
dnabert 5mer random
DNABERT _human _ft
DNABERT_ encRand _ft
DNABERT dataRand _ft
DNABERT _allRand _ft
CNN with different embeddings
DNABERT with different pre-trained models

4.1.2 TBFS motif occupancy identification (422)

AUPRC

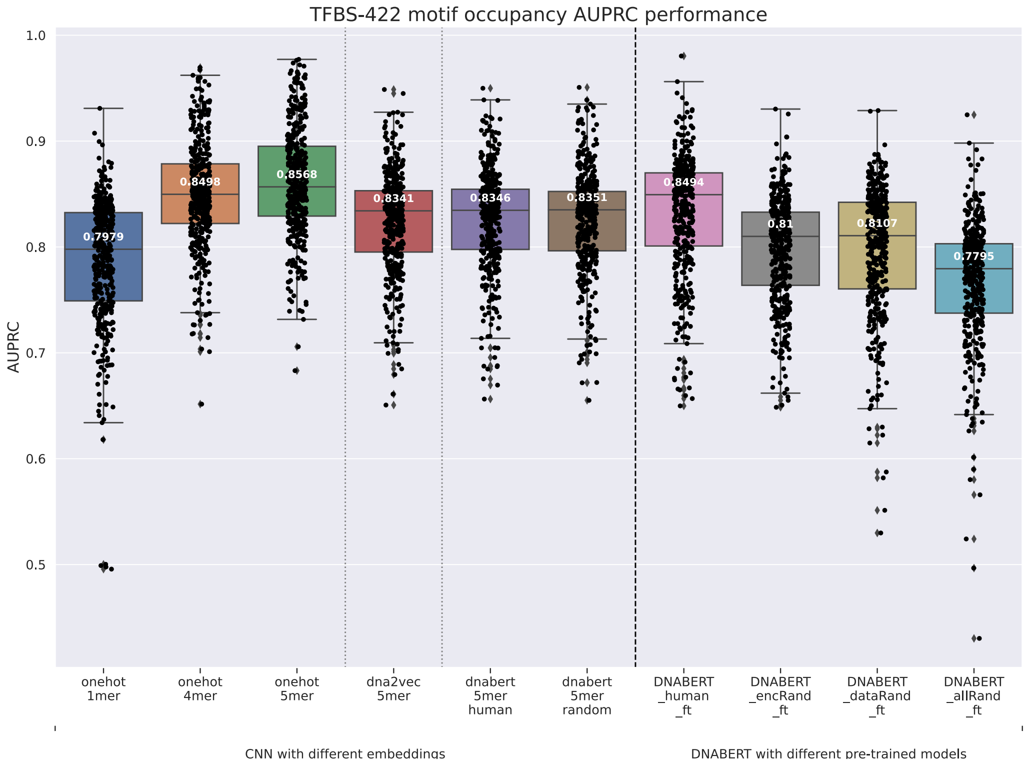

F1-score

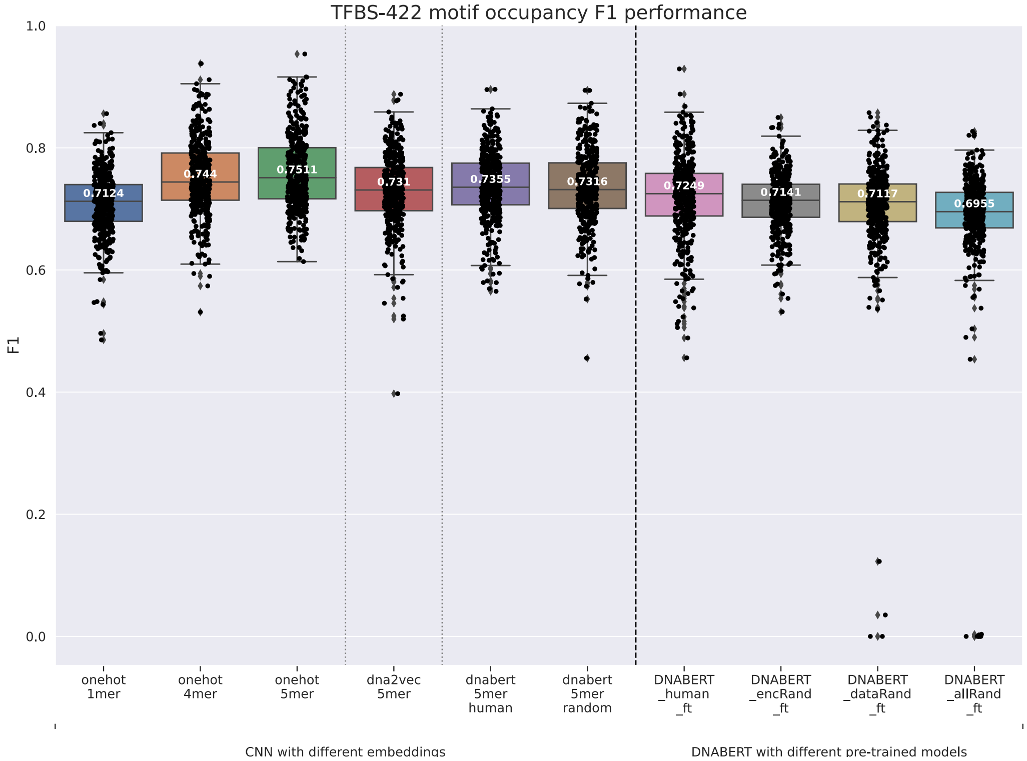

TFBS-422 motif occupancy F1 performance
F1
1.0
0.8
0.6
0.4
0.2
0.0
0.7124
0.744
0.7511
0.731
0.7355
0.7316
0.7249
0.7141
0.7117
0.6955
onehot 1mer
onehot 4mer
onehot 5mer
dna2vec 5mer
dnabert 5mer human
dnabert 5mer random
DNABERT _human _ft
DNABERT _encRand _ft
DNABERT _dataRand _ft
DNABERT _allRand _ft
CNN with different embeddings
DNABERT with different pre-trained models

MCC

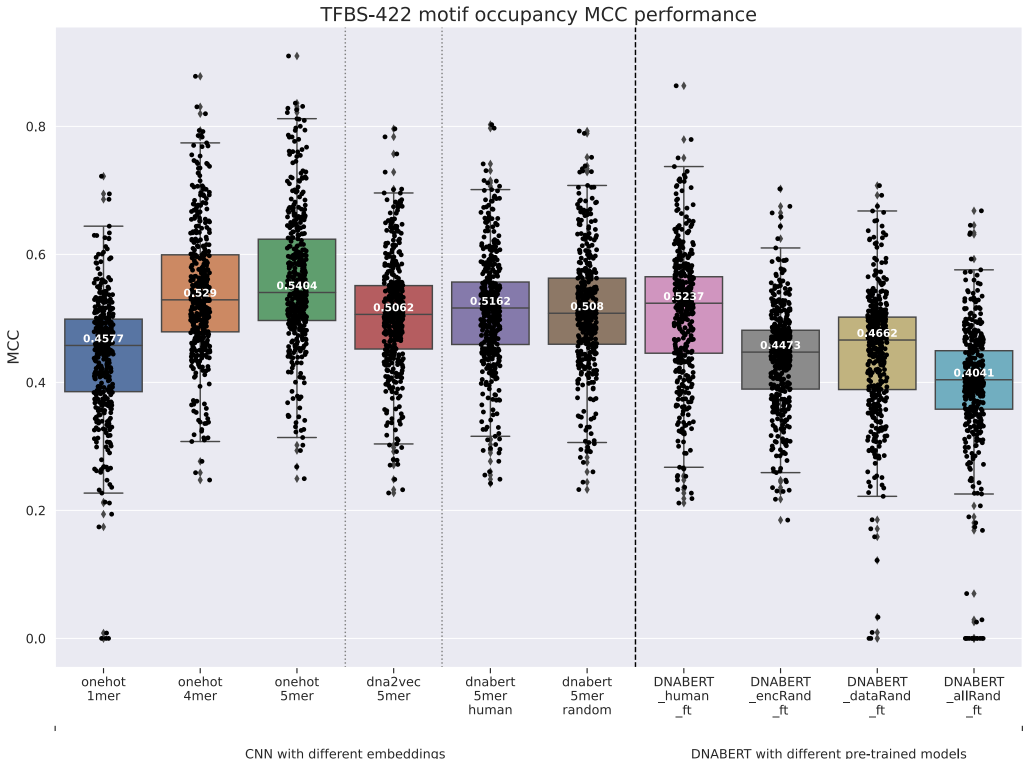

TFBS-422 motif occupancy MCC performance
MCC
0.0
0.2
0.4
0.6
0.8
0.4577
0.529
0.5404
0.5062
0.5162
0.508
0.5237
0.4473
0.4662
0.4041
onehot 1mer
onehot 4mer
onehot 5mer
dna2vec 5mer
dnabert 5mer human
dnabert 5mer random
DNABERT _human _ft
DNABERT _encRand _ft
DNABERT _dataRand _ft
DNABERT _allRand _ft
CNN with different embeddings
DNABERT with different pre-trained models

4.2 Additional experiment on other k-mer embeddings

According to reviewer's suggestion, we added other types of k-mer embeddings.

"

a. **For 5mer embedding, do not use 4^5 dimensional but rather use concatenated one-hot one-mer embedding with 4 * 5 = 20 dimensions.**
b. **To match the 100-dimensional dna2vec or 768-dimensional DNABERT embedding, I will recommend including a learnable embedding projected from the above-mentioned 20 dimensional embedding to 100 or 768 dimensions.**
c. **One-hot one-mer encoding with a first convolution layer with kernel size 5, input channels=4 and output channels=100, which outputs a learnable 100-dimensional 5-mer embedding.**

"

We implemented the five suggested embeddings, namely ***rev2vec1, rev2vec2_100, rev2vec2_768, rev2vec3_100*** and ***rev2vec3_768***. These embeddings are used as the input of the baseline encoder models. We added results on both TATA and TFBS tasks.

TATA promoter prediction task

TATA Human dataset

Embedding	Description	Number of parameters	AUROC	Precision	Recall	F1-score	MCC	AUPRC	ACC
Rev2vec1	1hot_concate	275534	0.9761	0.9380	0.9057	0.9216	0.8464	0.9781	0.9230
Rev2vec2_100	1hot_concate +linear(20,100)	1404914	0.9839	0.9446	0.9341	0.9393	0.8794	0.9841	0.9397
Rev2vec2_768	1hot_concate +linear(20,768)	18326690	0.9865	0.9388	0.9490	0.9439	0.8872	0.9878	0.9436
Rev2vec3_100	1hot_concate +conv(k=5,100)	1405170	0.9828	0.9475	0.9263	0.9368	0.8752	0.9840	0.9375
Rev2vec3_768	1hot_concate +conv(20,768)	18326946	0.9864	0.9610	0.9324	0.9465	0.8950	0.9876	0.9473
BERT_learned Human_5mer	k-mer pre-trained on human ref genome	18310562	0.9887	0.9465	0.9557	0.9511	0.9017	0.9886	0.9508
BERT_learned Random_5mer	k-mer pre-trained on random data	18310562	0.9883	0.9616	0.9398	0.9506	0.9026	0.9873	0.9512

TATA Mouse dataset

Embedding	Description	Number of parameters	AUROC	Precision	Recall	F1-score	MCC	AUPRC	ACC
Rev2vec1	1hot_concate	275534	0.9711	0.9123	0.9199	0.9161	0.8316	0.9739	0.9158
Rev2vec2_100	1hot_concate +linear(20,100)	1404914	0.9805	0.9415	0.9235	0.9324	0.8663	0.9821	0.9331
Rev2vec2_768	1hot_concate +linear(20,768)	18326690	0.9842	0.9682	0.9108	0.9386	0.8825	0.9867	0.9405
Rev2vec3_100	1hot_concate +conv(k=5,100)	1405170	0.9807	0.9319	0.9375	0.9347	0.8690	0.9835	0.9345
Rev2vec3_768	1hot_concate +conv(20,768)	18326946	0.9829	0.9487	0.9287	0.9386	0.8787	0.9842	0.9393
BERT_learned Human_5mer	k-mer pre-trained on human ref genome	18310562	0.9839	0.9491	0.9291	0.9390	0.8795	0.9849	0.9397
BERT_learned Random_5mer	k-mer pre-trained on random data	18310562	0.9860	0.9513	0.9426	0.9470	0.8945	0.9866	0.9472

TFBS prediction task

TFBS-690 dataset

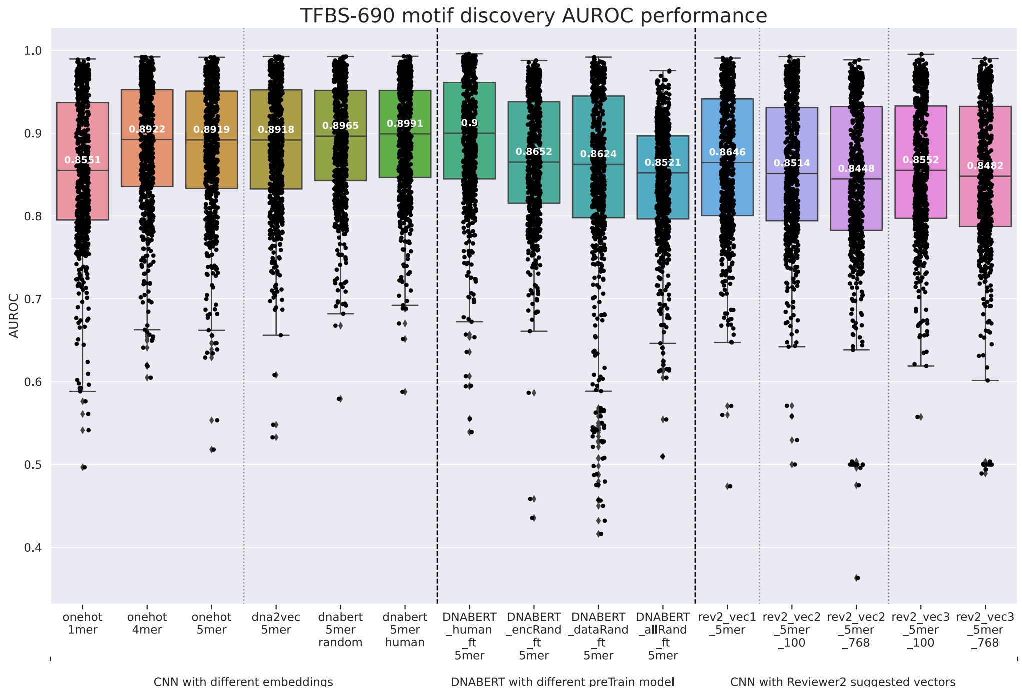

TFBS-422 dataset

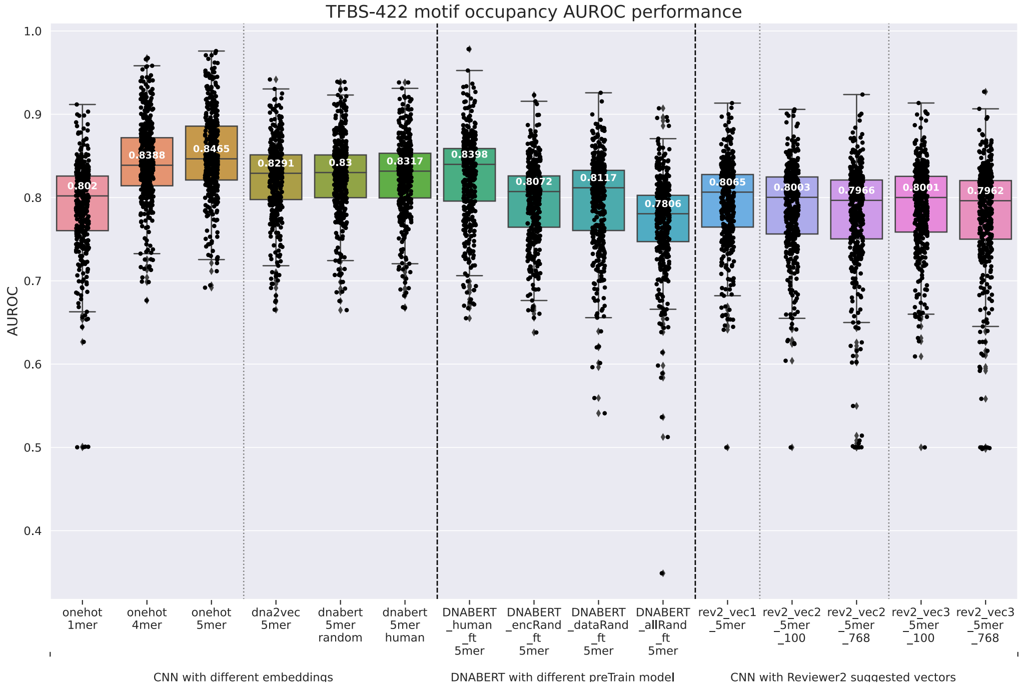

5. 1-mer DNABERT model

We analyzed of 1-mer models pre-trained on both human reference genome and randomly generated data. We examined the curve of validation loss for pre-trained models using a batch size of 2000. Validation losses were charted at 500 training-step intervals for each saved checkpoint. The y-axis denotes perplexity, calculated as exp(MLM_loss), where MLM_loss refers to the masked language modeling loss, equivalent to the cross entropy loss of masked tokens. The validation set derived from the human reference genome, is approximately 25% of the training data size.

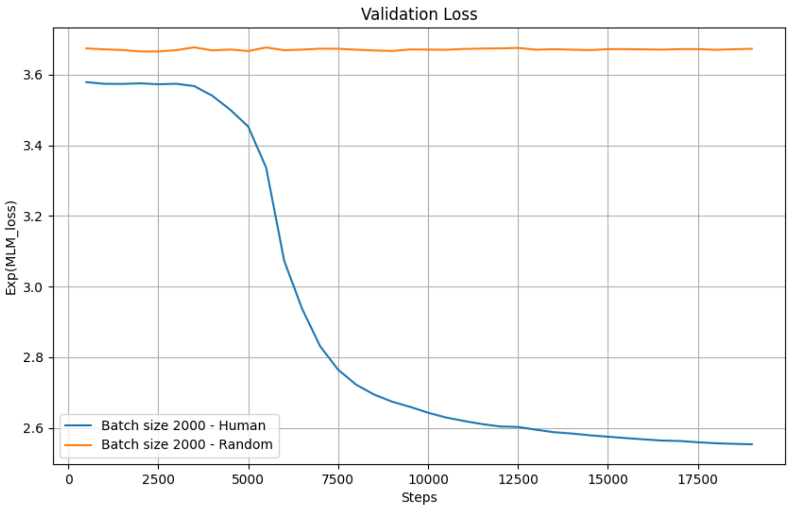

Figure S15 Validation loss for the 1-mer BERT model pre-trained on human reference data and random data.

As depicted in Figure S15, during the training phase, the validation loss of the DNABERT model pre-trained on human data starts to decline after 4,000 steps. In contrast, the validation loss of the model pre-trained on random data remains unchanged. We further investigated the loss in samples from the random training data specific to the random model. The loss values recorded were on a similar scale (~ 3.67) to those in the validation set. Given that the overlapping effect is eliminated in the 1-mer setting and random sequences lack inherent biological information, the non-convergence of the model pre-trained on random data aligns with our previous observations regarding overlapping k-mers.
